# Partitioning the Relative Importance of Phylogeny and Environmental Conditions on Phytoplankton Fatty Acids

**DOI:** 10.1371/journal.pone.0130053

**Published:** 2015-06-15

**Authors:** Aaron W. E. Galloway, Monika Winder

**Affiliations:** 1 John Muir Institute of the Environment, Watershed Science Center, University of California Davis, Davis, California, United States of America; 2 Department of Ecology, Environment and Plant Sciences, Stockholm University, Stockholm, Sweden; Texas A&M University at Galveston, UNITED STATES

## Abstract

Essential fatty acids (EFA), which are primarily generated by phytoplankton, limit growth and reproduction in diverse heterotrophs. The biochemical composition of phytoplankton is well-known to be governed both by phylogeny and environmental conditions. Nutrients, light, salinity, and temperature all affect both phytoplankton growth and fatty acid composition. However, the relative importance of taxonomy and environment on algal fatty acid content has yet to be comparatively quantified, thus inhibiting predictions of changes to phytoplankton food quality in response to global environmental change. We compiled 1145 published marine and freshwater phytoplankton fatty acid profiles, consisting of 208 species from six major taxonomic groups, cultured in a wide range of environmental conditions, and used a multivariate distance-based linear model to quantify the total variation explained by each variable. Our results show that taxonomic group accounts for 3-4 times more variation in phytoplankton fatty acids than the most important growth condition variables. The results underscore that environmental conditions clearly affect phytoplankton fatty acid profiles, but also show that conditions account for relatively low variation compared to phylogeny. This suggests that the underlying mechanism determining basal food quality in aquatic habitats is primarily phytoplankton community composition, and allows for prediction of environmental-scale EFA dynamics based on phytoplankton community data. We used the compiled dataset to calculate seasonal dynamics of long-chain EFA (LCEFA; ≥C_20_ ɷ-3 and ɷ-6 polyunsaturated fatty acid) concentrations and ɷ-3:ɷ-6 EFA ratios in Lake Washington using a multi-decadal phytoplankton community time series. These analyses quantify temporal dynamics of algal-derived LCEFA and food quality in a freshwater ecosystem that has undergone large community changes as a result of shifting resource management practices, highlighting diatoms, cryptophytes and dinoflagellates as key sources of LCEFA. Moreover, the analyses indicate that future shifts towards cyanobacteria-dominated communities will result in lower LCEFA content in aquatic ecosystems.

## Introduction

Aquatic habitats are increasingly stressed by fluctuation in key environmental variables, such as temperature and nutrients, that affect ecosystems, populations, and organisms [1–4]. Phytoplankton, which account for nearly half of net primary production on earth [5], are the fundamental producers of many complex biomolecules, including fatty acids in diverse lipid classes [6]. Fatty acids play critical roles in trophic interactions in aquatic food webs and for physiological processes in all organisms, including precursors to anti-inflammatory eicosanoids [7], maintenance of cell membrane function [6], and for energy storage (reviewed in [8]). Omega-3 (ɷ-3) and omega-6 (ɷ-6) poly-unsaturated fatty acids (PUFA) are synthesized almost exclusively by phytoplankton, macrophytes, and plants [8], and are critical classes of nutrients for diverse heterotrophs, including invertebrates, fish, and humans [9]. Because heterotrophs cannot synthesize ɷ-3 and ɷ-6 PUFA *de novo* [10], these molecules are considered to be ‘essential’ fatty acids (EFA) for consumers. Aquatic ecosystems are the primary source of ɷ-3 fatty acids in the biosphere, thus subsidizing both aquatic and terrestrial omnivores via the trophic transfer of these key EFA through food webs [11].

Phytoplankton growth and production is governed by nutrients, light, salinity, and temperature [12], and experiments have also shown that phytoplankton fatty acid composition is affected by these same variables (reviewed in [8,13]). Global climate change has resulted in increased surface temperatures of oceans [14], lakes [15], and rivers [16]. Increased water temperatures and alterations to water impoundment strategies can strengthen thermal stratification in coastal waters, and alter salinity regimes in lacustrine and marine habitats [17]. Land use changes and pollution by humans has resulted in increased nutrient deposition to both coastal [18] and inland water bodies [19,20]. Changing environmental conditions may therefore alter algal EFA content and food quality at the base of aquatic food webs, with consequences to both food webs and humans [21]. In addition, the importance of culture conditions on algal fatty acids is important for aquaculture practices, which often rely upon phytoplankton cultures for EFA critical for diverse larval fish and invertebrates [22].

Fatty acids are a promising biochemical constituent to use as a proxy for ecosystem-scale food quality because there is extensive literature documenting that growth and reproduction of diverse aquatic consumers are limited by certain EFA. While not all consumers are limited by the same EFA [10], there is extensive evidence that eicosapenaenoic acid (20:5ɷ3; EPA), arachidonic acid (20:4ɷ6; ARA), and docosahexaenoic acid (20:5ɷ3; DHA), collectively defined here as long-chain EFA (LCEFA; e.g., ≥C_20_ ɷ-3 and ɷ-6 PUFA), are exceptionally important for diverse aquatic organisms [9]. For example, experiments have identified EPA as a strong predictor of growth in cladoceran zooplankton [23,24], and isopods [25]. Increased dietary arachidonic ARA correlates with improved growth in juvenile bivalves [26], gonad development in sea urchins [27–29], and higher fecundity in marine copepods [30]. DHA is critical to fish larval development [31–33], and copepod egg viability [34], and egg-production rates [35]. High quality diets, such as those that are rich in LCEFA content, result in faster growth or higher reproductive rates for zooplankton and fish [36–38]. Ecosystems may be thus constrained by the abundance of LCEFA in the environment [21,39,40].

The value of essential nutrients often propagates up the food chain; for example, multiple species of larval fish raised on diets of rotifers which were themselves fed on EFA rich diets exhibited higher growth and survival than fish fed with rotifers fed EFA poor diets [33,36]. Larval herring in the field have their highest RNA/DNA derived growth rates during periods of high DHA concentration in the Kiel Canal [41]. Food web models have shown that increased abundance of high quality phytoplankton can enable high growth efficiency for zooplankton predators, in turn allowing for subsequently high zooplanktivory by fish [42]. The presence of particularly EFA-rich phytoplankton may therefore result in the highly efficient transfer of energy through aquatic food webs, resulting in inverted biomass distributions due to the ability of fast growing zooplankton populations to withstand high predation by fish [43]. Because of the importance of these essential molecules at all trophic levels, including the importance of subsidies of EFA from aquatic to terrestrial environments [11], creative approaches for monitoring and estimating ecosystem-scale EFA content in the real world are needed.

The fatty acid composition of algae is determined by both phylogenetic affiliation and environmental characteristics [8], but the relative importance of each of these factors on algal fatty acids has not been quantified. Analyses of diverse algal species from lab studies [8,44,45] and seston in field studies [46–50] have shown that fatty acid composition is largely explained by the phylogenetic relationships of phytoplankton. However, experiments within particular algal groups that have manipulated environmental variables have also shown that algal fatty acids are sensitive to culture conditions (reviewed in [8,13,39]), particularly temperature, light, and nutrients [51–53]. Phytoplankton community composition is a strong predictor of fatty acid content in nature; for example, phytoplankton abundance explained 79% of the variation in total seston fatty acid concentration in a diverse marine dataset [49]. Indeed, it has recently been shown that it is possible to quantitatively infer phytoplankton abundance in lakes from seston fatty acids [54]. Moreover, researchers have also recently estimated concentrations of particular EFA from lake seston community composition [46,55] and satellite-derived estimates of open ocean diatom abundance [21]. Because the EFA content of resources is a key determinant of their quality as food for heterotrophs [56], it is important to understand the mechanisms that control phytoplankton fatty acid composition. A comparative assessment of these factors in determining algal food quality is necessary in order to anticipate whether changing environmental conditions will drive basal food quality directly, through physiological controls, or indirectly, through changes in phytoplankton community composition due to physical mixing, algal competition, or trophic pressures (e.g., [57]).

We synthesized a large dataset of phytoplankton fatty acid profiles, consisting of more than 200 species from six major taxonomic groups, which were cultured under diverse conditions, to address three general questions; 1) what is the relative importance of ‘nature’ (taxonomic affiliation) versus ‘nurture’ (manipulated culture conditions) for phytoplankton fatty acid composition; 2) what are the relationships between LCEFA and the top-ranked culture condition variables; and 3) does sum of LCEFA, as a proxy for food quality, of these diverse phytoplankton groups differ? The results indicate that taxonomic composition of phytoplankton is the primary determinant to food quality in aquatic food webs, implying that environmental change will therefore affect basal food quality indirectly, via affects to phytoplankton community composition. To put these results in an ecological context, we used the compiled fatty acid data to calculate algal-derived LCEFA concentrations and ɷ-3:ɷ-6 LCEFA ratios as indicators of primary producer food quality in a freshwater ecosystem using existing multi-decadal phytoplankton time series. These results show how changes in algal community composition drive LCEFA concentration in aquatic ecosystems at seasonal and annual time scales.

## Materials and Methods

### Phytoplankton fatty acids

We conducted a synthesis of marine and freshwater phytoplankton fatty acid literature, initially compiling 1145 published profiles from 208 species in 13 taxonomic groups (roughly defined by Class; details in Supporting Information). We used ISI Web of Science searches including the terms ‘fatty acids’, ‘phytoplankton’ and each of eight major phytoplankton group names [Chlorophyta; Cryptophyta; Cyanobacteria; Bacillariophyceae, Coscinodiscophyceae, Fragilariophyceae (e.g., all Diatoms); Dinophyta, Haptophyta], to identify potential data sources. We also identified additional potential datasets using a combination of prior knowledge of the literature and through tracing the citations of other key studies in the field. The justification for this varied approach is that phytoplankton fatty acid profiles are reported throughout a diverse literature with very different original study goals. As a result of this literature search we screened 399 papers, including phycological surveys and experimental trophic studies. We built a new fatty acid database from the published data from 58 of these studies (S1 and S2 Tables, S1 Dataset), which fulfilled basic data consistency requirements explained in further detail in the Supporting Information (S1 File). We did not exclude studies that we perceived might report ‘outlier’ fatty acid profiles based on extreme experimental manipulation of conditions expected to affect fatty acid composition. The decision to exclude papers from the synthesis was based only on whether papers met our *a priori* criteria, and does not imply actual or perceived flaws in the original papers.

Briefly, we included data only from studies for which information on the following culture conditions were either available in the paper or could be obtained from the authors: irradiance (μ Mol m^-2^ s^-1^), hours of light per day, temperature (°C), salinity (parts per thousand; ppt), and a single binary indicator of nutrient status or growth phase (termed ‘nutrient status’ in the analysis). We therefore assumed that cultures in exponential growth were not nutrient limited and that ‘stationary’ cultures were nutrient limited. We standardized the various units for each measurement or environmental condition used by the original authors into consistent units (see S1 File). When such standardizations could not be made from the information in the paper or through communication with the authors, we did not include data from those sources. Researchers do not always report the same fatty acids for various reasons. To include data from as many studies as possible, we initially recalculated each published fatty acid profile to comprise of 11 consistent individual fatty acids or categories. The 8 individual ɷ-3 and ɷ-6 EFA are: 18:2ɷ6 (LIN), 18:3ɷ6 (GLA), 18:3ɷ3 (ALA), 18:4ɷ3 (SDA), 18:5ɷ3, 20:4ɷ6 (ARA), 20:5ɷ3 (EPA), and 22:6ɷ3 (DHA). In addition, we compiled 3 summary fatty acid categories for each profile, including; sum of saturated fatty acids (SAFA), sum of monounsaturated fatty acids (MUFA), and sum of non-EFA PUFA, or ‘other PUFA’. The other PUFA variable was calculated by subtracting the total of the 8 individual EFA from the total remaining PUFA in the original datasets. This ensured that the analytical dataset did not have double-reported variables. The other PUFA variable mostly captures the sum of C_16_ PUFA and other relatively rare >C_18_ PUFA.

The units that researchers use for fatty acid data are not consistent and can depend on the original goals or analytical methods. Our analyses therefore focused on the most common formats, percentage of total fatty acids (% FA), and fatty acids as a percentage of algal dry weight (FA % DW). The majority of the fatty acid profiles (n = 730) analyzed here were either originally reported as % FA, or were converted to this format from the original concentration units used by the original authors. Because the absolute quantity of particular fatty acids may be the ideal format to consider from a food quality perspective [39,52,53], our analyses also used the most commonly reported form of fatty acid concentration data measured for diverse phytoplankton groups (n = 116 FA % DW profiles). Other common fatty acid concentration data formats were compiled (S1 Dataset) but were not analyzed here due to low numbers of unique fatty acid taxa profiles within each group. Extensive discussion of criteria for inclusion, detection limits, conversion of units, data format, and examples of the reasoning applied to specific research is provided in the Supporting Information (S1 File).

### Partitioning variation in phytoplankton fatty acids

We used principal components analysis (PCA) to visualize arcsine-square root transformed multivariate fatty acid signatures of the six major algal taxonomic groups (see S2 Table) in the larger % FA dataset that are the focus of the vast majority of the published studies in all culture conditions (n = 666 profiles, all culture conditions). The PCA was calculated using the prcomp function in R [58]. To evaluate the significance of the apparent phytoplankton group separation observed in the PCA, we used a one-way permutational multivariate analysis of variance (PERMANOVA [59]) on the same fatty acid dataset (Euclidean distance, 9999 permutations, with the adonis function in R).

We used a distance-based linear model (DISTLM [60]), an extension of distance-based redundancy analysis (dbRDA [61]), to quantify the relative contribution of algal group affiliation and multiple culture condition variables on multivariate algal fatty acid composition. DISTLM allows for partitioning the variance explained by a combination of categorical and continuous ‘explanatory’ variables (**X** matrix) on a multivariate dataset (response variables; **Y** matrix), in the form of a Euclidean distance matrix. The explanatory matrix consisted of the raw continuous variables (i.e., culture conditions) and two groups of categorical variables (taxonomic group and nutrient status), which were coded as a series of individual binary variables and grouped as common indicator variable ‘sets’ prior to running DISTLM in PERMANOVA+ for Primer [60].

The DISTLM was run on each of two distinct arcsine-square root transformed fatty acid datasets (% FA and FA % DW), using the same six major algal taxonomic groups (S2 Table). Only profiles with all explanatory variables were included in this analysis, resulting in datasets of 621% FA and 109 FA % DW observations, respectively. We used a step-wise selection procedure (9999 permutations) with adjusted *R* ^2^ as the selection criterion, and report results of marginal tests as well as total variation explained by each variable. The full model is visualized with a dbRDA, which shows results of fitting the model first to the most important variable and sequentially partitioning the remaining unexplained variation among remaining variables.

### Phytoplankton long chain essential fatty acids

We calculated the sum of the long-chain essential ɷ-3 and ɷ-6 fatty acids (LCEFA); defined here as docosahexaenoic acid (DHA; 22:6ɷ3), eicosapentaenoic acid (EPA; 20:5ɷ3), and arachidonic acid (ARA; 20:4ɷ6) for both the FA % DW and % FA datasets. We performed post-hoc correlations (Kendall’s Tau) between this univariate fatty acid summary category and each of continuous culture condition variables that explained more than 5% of the variation in the full fatty acid dataset DISTLM marginal tests.

We defined a fatty acid-based food quality index (FQI) based on the composition of ɷ LCEFA at the algal group level [62]. We first calculated the average Σ LCEFA content of each unique algal taxa, across all culture conditions, then calculated the average group Σ LCEFA from the unique taxa means within each group. First aggregating at the species level removed potential oversampling bias from calculating group averages from all raw data, which include many profiles of highly studied taxa. The scale of the index (0–1; Eq 1) was defined by calculating the relative quality of each algal group (AG) *i* compared to the maximal LCEFA content of all AG:

$${\text{FQI AG}}_{i}=\;\left(\frac{{\text{AG}}_{i}\;\text{LCEFA}}{\text{maximum LCEFA all AG}}\right)$$ (1)

Where the FQI for each algal group (AG_*i*_) is the average Σ LCEFA of AG_*i*_ divided by the maximum Σ LCEFA of all groups. We applied this calculation to both % FA and FA % DW datasets to compare the relative LCEFA ranking among phytoplankton groups between the two fatty acid data types that are most commonly reported in the literature.

### Ecosystem algal derived long chain essential fatty acid dynamics

We used the calculated average Σ LCEFA, ɷ-3, and ɷ-6% DW content of each unique algal group to calculate algal-derived average concentrations of these three fatty acid categories from phytoplankton biomass in a natural system (Eq 2):

$$\sum \mu \text{g FA category}\;{\text{L}}^{-1}=\sum_{i=1}^{\#\text{AG}}\mu \text{g biomass}\;{\text{AG}}_{i}*\;\left(\frac{\text{average}\;{\text{AG}}_{i}\;\text{FA}\;\%\;\text{DW}}{100}\right)$$ (2)

Where, for each fatty acid category (Σ LCEFA, ɷ-3, and ɷ-6), the total calculated μg FA L^-1^ is the sum across the major algal groups (#AG = 6) of the total biomass (μg C L^-1^) of AG_*i*_ in the field multiplied by the average fatty acid content of AG_*i*_. The AG_*i*_ FA % DW data is first divided by 100 to express it as a proportion before it is multiplied by the AG_*i*_ biomass.

We performed this calculation using freshwater phytoplankton data from Lake Washington (1961–2001) (e.g., [63]), as a case study of LCEFA seasonal dynamics. We selected this system as an example because there is a monthly-sampled 40-year phytoplankton time series [63,64] which documents a large phytoplankton community transition. Lake Washington is a large (surface area ca. 88 km^2^, mean depth ca. 33 m), seasonally stratified lake [63], which has undergone significant changes in trophic structure due largely to eutrophication prior to the mid-1970s (reviewed in [63,65,66]). We defined two multi-year phases for comparative analysis in the 1961–2001 Lake Washington data: 1961–1969, which was the period of maximal raw sewage input into the lake [65]; and 1975–2001, a clear-water phase [63], indicating a period of recovery from the raw sewage input and plankton community changes [66,67]. Average monthly phytoplankton biomass for all years (μg C L^-1^) was calculated from the original reported biovolume data (μm^3^ mL^-1^) following equations for diatoms and non-diatom phytoplankton reported in ref. [68]. The monthly ɷ-3:ɷ-6 ratios, as an indicator or potential food quality for zooplankton, are the total calculated μg ɷ-3 L^-1^ divided by the total calculated μg ɷ-6 L^-1^ from Eq 2. While specific optima of dietary content of LCEFA or ɷ-3:ɷ-6 fatty acid ratios may not be consistent for all zooplankton [69], optimal dietary ɷ-3:ɷ-6 fatty acid ratios for filter feeding cladocerans are ≥3 (*Daphnia galeata*), and ≥5 (*Daphnia magna*) (reviewed in [69]).

## Results

### Partitioning variation in phytoplankton fatty acids

Phytoplankton groups were clearly separated (PERMANOVA; MS = 13.28, *F*_5_ = 95.16, *p* = 0.0001) by their multivariate fatty acid profiles (11 fatty acids, arcsine-square root transformed; S1 Fig). PCs 1 and 2 accounted for 51% of the total variation and PC3 accounted for 15%. A plot of Pearson correlations between the FA variables and PCA scores (S1 Fig) identified a strong negative correlation between EPA (20:5ɷ3) and PC1, which was an area of the PCA largely dominated by diatoms. PC1 was positively correlated with α-linolenic acid (18:3ɷ3) and linoleic acid (18:2ɷ6), the region of the PCA dominated by chlorophytes, Cyanobacteria and some cryptophytes. The SAFA and MUFA were negatively correlated with PC2, corresponding particularly to Cyanobacteria and diatoms. PC2 was positively correlated with nearly all ɷ-3 fatty acids, particularly stearidonic acid (SDA; 18:4ɷ3), and corresponded to region of the ordination dominated by cryptophytes. Haptophytes and dinoflagellates were not differentiated from other groups in PC1 or PC2 but were separated from other algae by PC3 (not pictured). The positive axis of PC3 was positively correlated with several ɷ-3 fatty acids, particularly DHA (22:6ɷ3). The negative axis of PC3 was correlated with the ‘other PUFA’, which mostly consists of C_16_ PUFA, and was dominated by diatoms and chlorophytes.

The marginal tests in the DISTLM analysis (S3 Table) report the proportion of the variation explained by each variable in each dataset, independent of other explanatory variables. For the % FA dataset, taxonomic group explained 43.6% of the total variation, with the next most important factor being salinity, which explained 11.0% of the total. Other culture condition variables explained little variance (all <3.3%). For the FA % DW dataset, group accounted for 36.2% of the variation and the second most important factor was ‘hours light’, which explained 11.2% of the total. The proportions of the variance explained by each of the remaining culture condition variables were generally much higher in the FA % DW dataset compared with the % FA data (between 3.1–7.1%; S3 Table). The results of the sequential step-wise test using adjusted *R* ^2^ selection criterion procedure for each dataset are shown in Table 1. The cumulative variation explained by the full model was 48.4% for the % FA data and 56.8% for the FA % DW data (Table 1). In the % FA data, addition of the five culture conditions to taxonomic group increased the cumulative variation explained by 4.8%, whereas the addition of the culture conditions to group in the FA % DW dataset increased the cumulative variation explained by 20.6%.

**Table 1:** Results of the DISTLM sequential step-wise tests.

Dataset	Variable	Adjust. *R* ^2^	SS(tr.)	Pseudo-*F*	*P*	prop. var.	cum. var.	res. df	regr. df
% FA	Group	0.432	66.40	95.16	0.0001	0.436	0.436	615	6
	Salinity	0.453	3.29	24.50	0.0001	0.022	0.458	614	7
	Light	0.465	1.98	15.08	0.0001	0.013	0.471	613	8
	Nutrient	0.469	0.82	6.28	0.0001	0.005	0.476	612	9
	Temp	0.473	0.65	5.03	0.0003	0.004	0.481	611	10
	Hrs. Light	0.475	0.46	3.59	0.0016	0.003	0.484	610	11
FA % DW	Group	0.331	1.71	11.67	0.0001	0.362	0.362	103	6
	Light	0.403	0.35	13.51	0.0001	0.075	0.436	102	7
	Hrs. Light	0.447	0.22	9.11	0.0001	0.047	0.483	101	8
	Nutrient	0.493	0.23	10.22	0.0001	0.048	0.531	100	9
	Salinity	0.519	0.13	6.30	0.0001	0.028	0.559	99	10
	Temp	0.523	0.04	1.94	0.0793	0.009	0.568	98	11

Results of distance-based linear model (DISTLM) sequential step-wise tests using adjusted *R* ^*2*^ selection criterion for both fatty acid datasets (% FA and FA % DW). This test reports the proportion of the variation explained (prop. var) and the cumulative variation (cum. var) explained by the model at each step. Variable abbreviations: nutrient status [Nutrient (replete or limited)]; light intensity (Light); hours of light (Hrs. Light); temperature (Temp).

**Fig 1:**
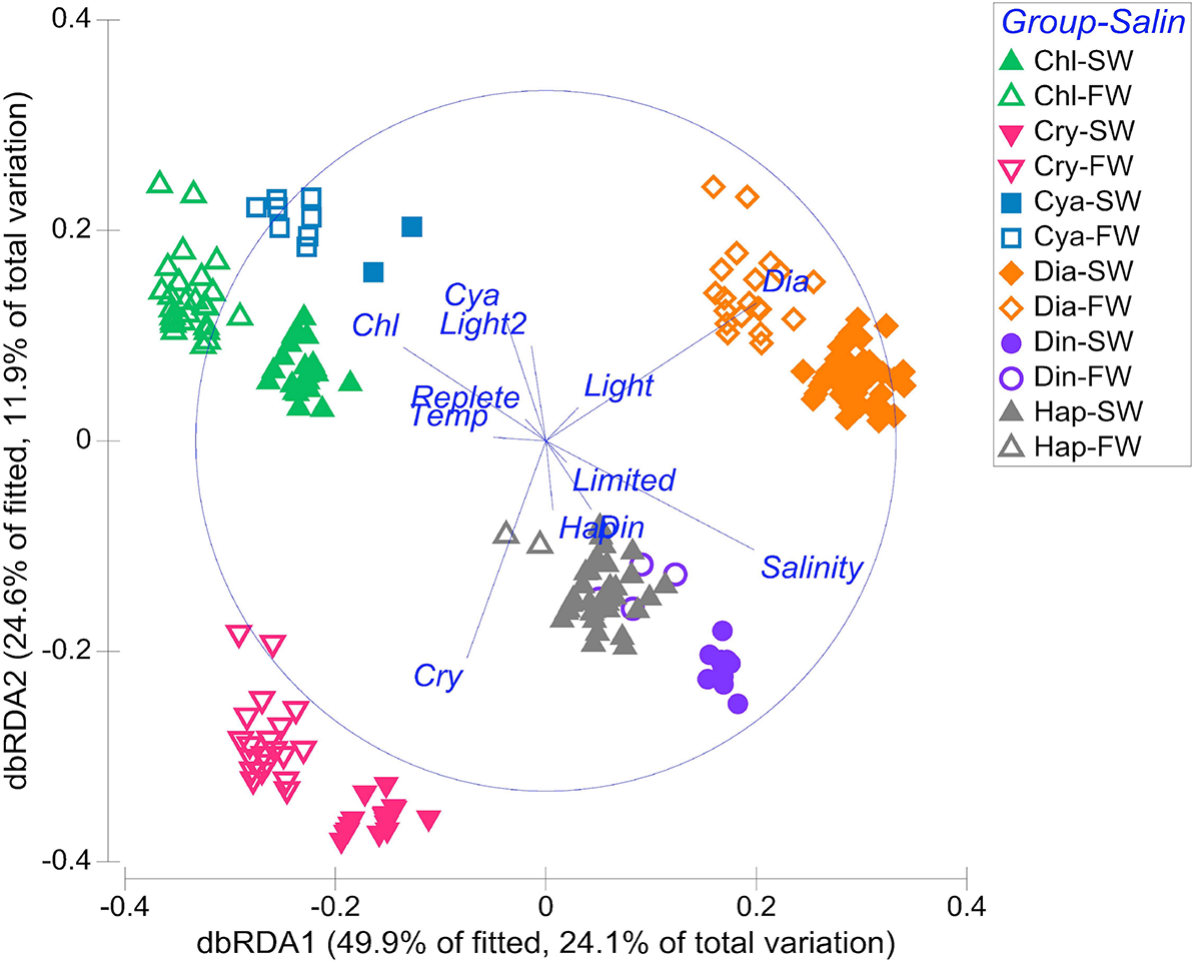
Distance-based redundancy analysis (dbRDA) ordination of the full multivariate ISTLM. The DISTLM partitioned the variance in phytoplankton fatty acids explained by the predictor variables for all 621 phytoplankton % FA profiles for which all culture conditions were available [follows Table 1 variable descriptions except salinity here is coded as freshwater (FW) or saline water (SW) for visualization)]. The group abbreviations are: Chl (Chlorophyta); Cry (Cryptophyta); Cya (Cyanobacteria); Dia [diatoms (Bacillariophyceae, Coscinodiscophyceae, Fragilariophyceae)]; Din (Dinophyta); and Hap (Haptophyta). The first two axes explained 75% of the variability in the fitted model. The top two ranked variables in the model for this dataset, group and salinity, are identified with the symbols. Vector overlays show the strength of the relationship between the predictor variables and the dbRDA axes.

The full model of the % FA dataset is visualized with a dbRDA where the symbols and colors are coded to identify the two most important variables in the model (Fig 1), group and salinity. The first two dbRDA axes captured 74.1% of the variability of the fitted model and 36% of the variation in the entire fatty acid dataset. Relative to freshwater taxa, saltwater strains of each algal group are all oriented towards the positive axis of dbRDA1 and negative axis of dbRDA2. The vectors show the relationships of the dbRDA axes on each of the explanatory variables, including the categorical variables of group and nutrient status. All algal groups clearly separated from each other except haptophytes and dinoflagellates, and chlorophytes and cyanobacteria grouped in the same quadrant of the plot, in the negative axis of dbRDA1 and the positive axis of dbRDA2.

### Phytoplankton long chain essential fatty acids

**Fig 2:**
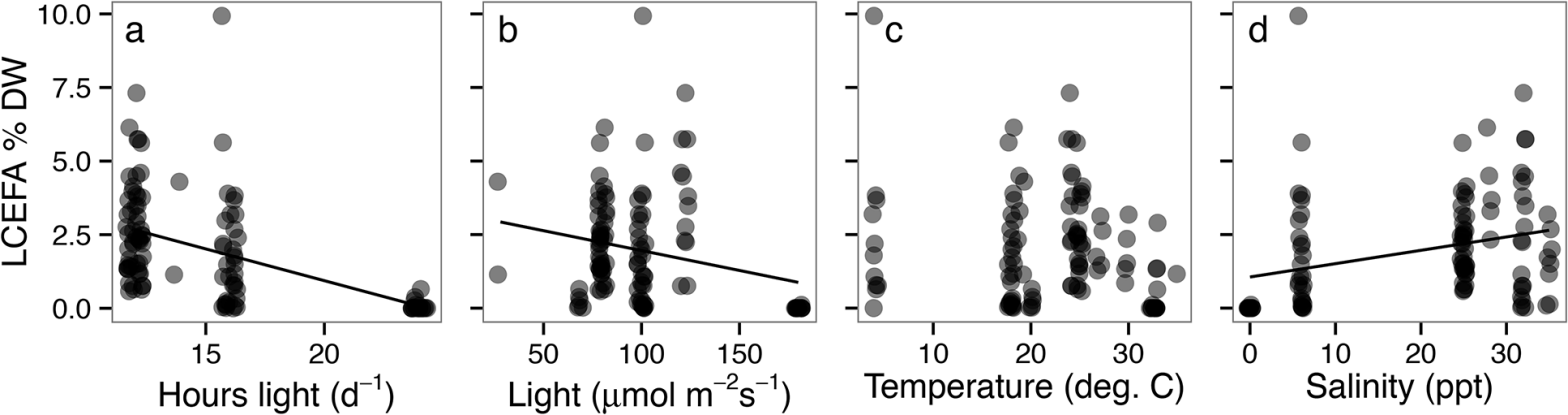
Post-hoc correlations between continuous culture conditions and the Σ LCEFA in the % DW dataset. Panels: (a) hours light, (b) light intensity, (c) temperature, and (d) salinity for all phytoplankton groups pooled together. Each point is from a different % DW fatty acid profile (i.e., not species means), and all points are the same tone of grey, but are plotted with semi-transparency and jittering to accommodate for overplotting of points with similar values. Best-fit lines are significant (Kendall’s Tau, p<0.05).

**Fig 3:**
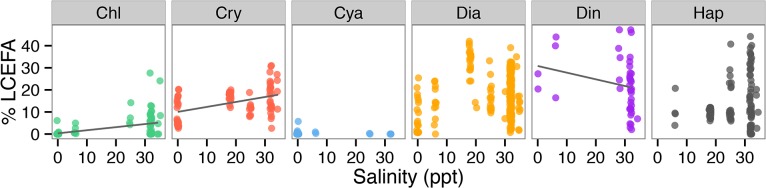
Phytoplankton group specific correlations between the Σ LCEFA in the % FA dataset and salinity of culture conditions. Salinity and % LCEFA was positively correlated (Kendall’s Tau, p<0.001) for chlorophytes and cryptophytes, negatively correlated for dinoflagellates, and not correlated for the other groups. Group abbreviations follow Fig 1. All points within each algal group are the same color, but are plotted with semi-transparency to accommodate for over plotting of points with similar values. Separate correlations for each phytoplankton group were performed due to the larger within-group sample sizes in this dataset.

Post-hoc analyses of the Σ LCEFA for all phytoplankton profiles (i.e., not species means) and the most important culture condition variables from the DISTLM marginal tests, identified negative correlations between LCEFA % DW and both hours light (Kendall’s Tau = -0.494, n = 109, p<0.0001; Fig 2A) and light intensity (Tau = -0.158, n = 109, p = 0.0300; Fig 2B). The negative relationship between light intensity and total algal LCEFA % DW was not significant when a post-hoc analysis was run without the 12 cyanobacterial strains that were cultured in the rather extreme light intensity of 180 μmol m^-2^ s^-1^. The relationship between the total h of light d^-1^ and total LCEFA % DW was still significant when the 18 profiles from species cultured in 24 hours of light d^-1^ were removed (Kendall’s Tau = -0.263, n = 91, p = 0.002). There was slight negative but non-significant relationship between LCEFA % DW and temperature (Tau = -0.121, n = 109, p = 0.079, Fig 2C). LCEFA % DW and salinity were positively correlated (Tau = 0.310, n = 109, p<0.0001; Fig 2D). Correlations between the % LCEFA dataset and salinity of culture conditions were performed for each phytoplankton group due to the larger within-group sample sizes in this dataset. Salinity and % LCEFA (Fig 3) was positively correlated for chlorophytes (Tau = 0.553, n = 129, p<0.0001) and cryptophytes (Tau = 0.331, n = 87, p<0.0001), and negatively correlated for dinoflagellates (Tau = -0.332, n = 41, p = 0.0080).

**Fig 4:**
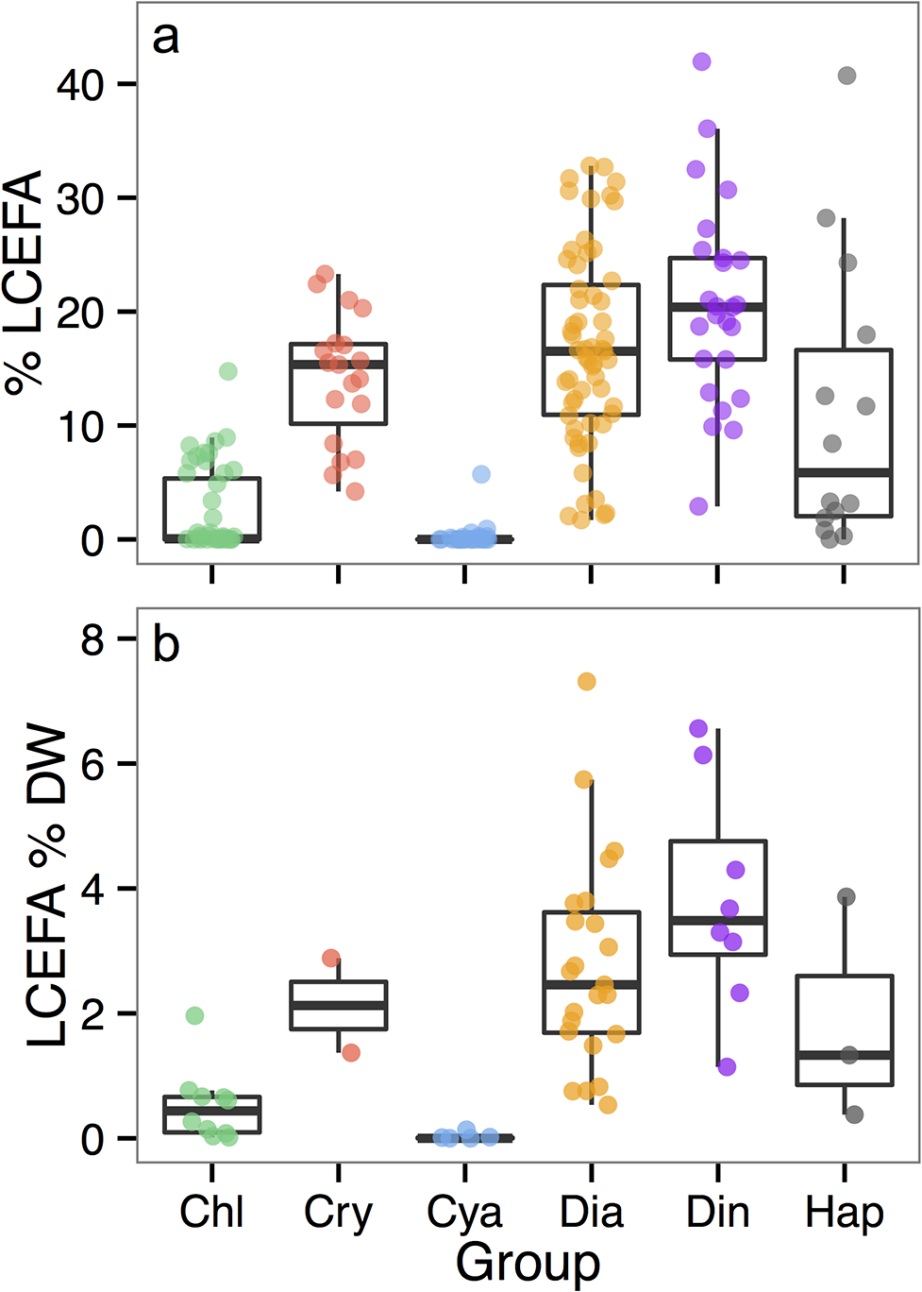
Boxplots of species averages of Σ long-chain essential fatty acids (LCEFA) in six major phytoplankton groups. (a) Shows the % FA dataset, consisting of 208 averages from 666 raw profiles. (b) Shows the FA % DW dataset, consisting of 55 averages from 105 raw profiles. Group name abbreviations follow Fig 1. The heavy line is the median, box boundaries are the 25^th^ and 75^th^ percentiles, and whiskers extend to the most extreme value within 1.5*IQR (interquartile range). The y-axis is set to show the extent of whiskers, thus some extreme outliers are not plotted (outliers were included in calculation of average group LCEFA).

The calculated species averages of Σ LCEFA in each dataset, split by phytoplankton group, are plotted in Fig 4, and summary statistics for Σ LCEFA, ɷ-3, and ɷ-6 fatty acids are in Table 2. LCEFA values in dinoflagellates, diatoms and cryptophytes ranged between ca. 16–20% of total % FA and ca. 2–4% of total DW. Total LCEFA was intermediate in haptophytes and chlorophytes and uniformly low or non-existent in cyanobacteria. The relative FQI of phytoplankton groups, calculated from the LCEFA content in each group following Eq 1, was similar between the two datasets (S2 Fig, Table 2).

**Table 2:** Summary of % LCEFA, % ɷ-3, and % ɷ-6 by phytoplankton group and dataset.

			LCEFA	LCEFA	LCEFA	ɷ-3	ɷ-3	ɷ-6	ɷ-6
Group	Dataset	species N	mean	sd	FQI	mean	sd	mean	sd
Chl	% FA	47	2.28	3.62	0.11	31.77	9.97	8.67	5.57
Cry	% FA	19	14.13	5.70	0.65	54.32	12.14	6.65	5.58
Cya	% FA	43	0.19	0.88	0.01	16.33	11.29	13.51	11.05
Dia	% FA	59	16.80	8.49	0.77	17.41	10.20	4.06	3.33
Din	% FA	26	21.69	10.09	1.00	40.61	13.97	2.65	2.07
Hap	% FA	14	11.13	12.50	0.51	22.88	16.07	6.22	5.38
Chl	FA % DW	10	0.52	0.58	0.14	3.14	1.81	1.06	0.65
Cry	FA % DW	2	2.13	1.07	0.56	9.70	4.25	0.32	0.16
Cya	FA % DW	9	0.02	0.04	0.00	1.96	1.08	1.47	0.86
Dia	FA % DW	23	2.77	1.67	0.73	2.26	1.23	1.51	1.37
Din	FA % DW	8	3.82	1.82	1.00	6.27	3.00	0.54	0.55
Hap	FA % DW	3	1.86	1.80	0.49	5.22	2.67	1.12	0.83

Phytoplantkon group mean and standard deviation (sd) for each fatty acid category (% LCEFA, % ɷ-3, and % ɷ-6) and a relative food quality index (FQI) based on total LCEFA content, calculated across unique species (e.g., species N, not all raw fatty acid profiles) in each dataset.

### Ecosystem algal derived long chain essential fatty acid dynamics

**Fig 5:**
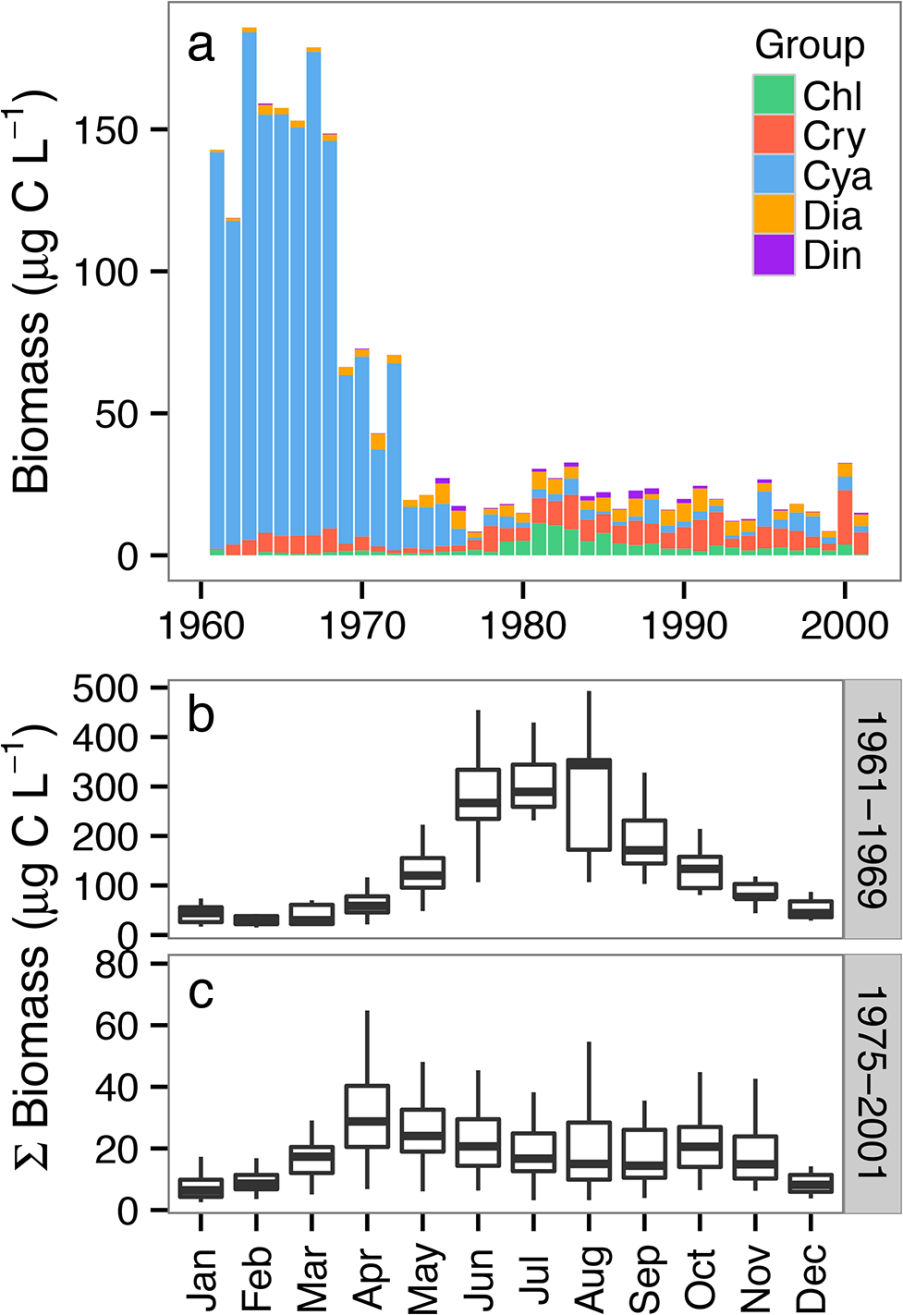
Lake Washington annual and monthly phytoplankton biomass from 1961–2001. (a) Stacked barplots of annual average phytoplankton biomass, color-coded by taxonomic group, from 1961–2001. Boxplot of average total phytoplankton biomass during the (a) eutrophic years (1961–1969) prior to sewage treatment, and (b) the oligotrophic years (1975–2001) after the transition (1970–1974) to sewage treatment in Lake Washington. Abbreviations follow Fig 1.

**Fig 6:**
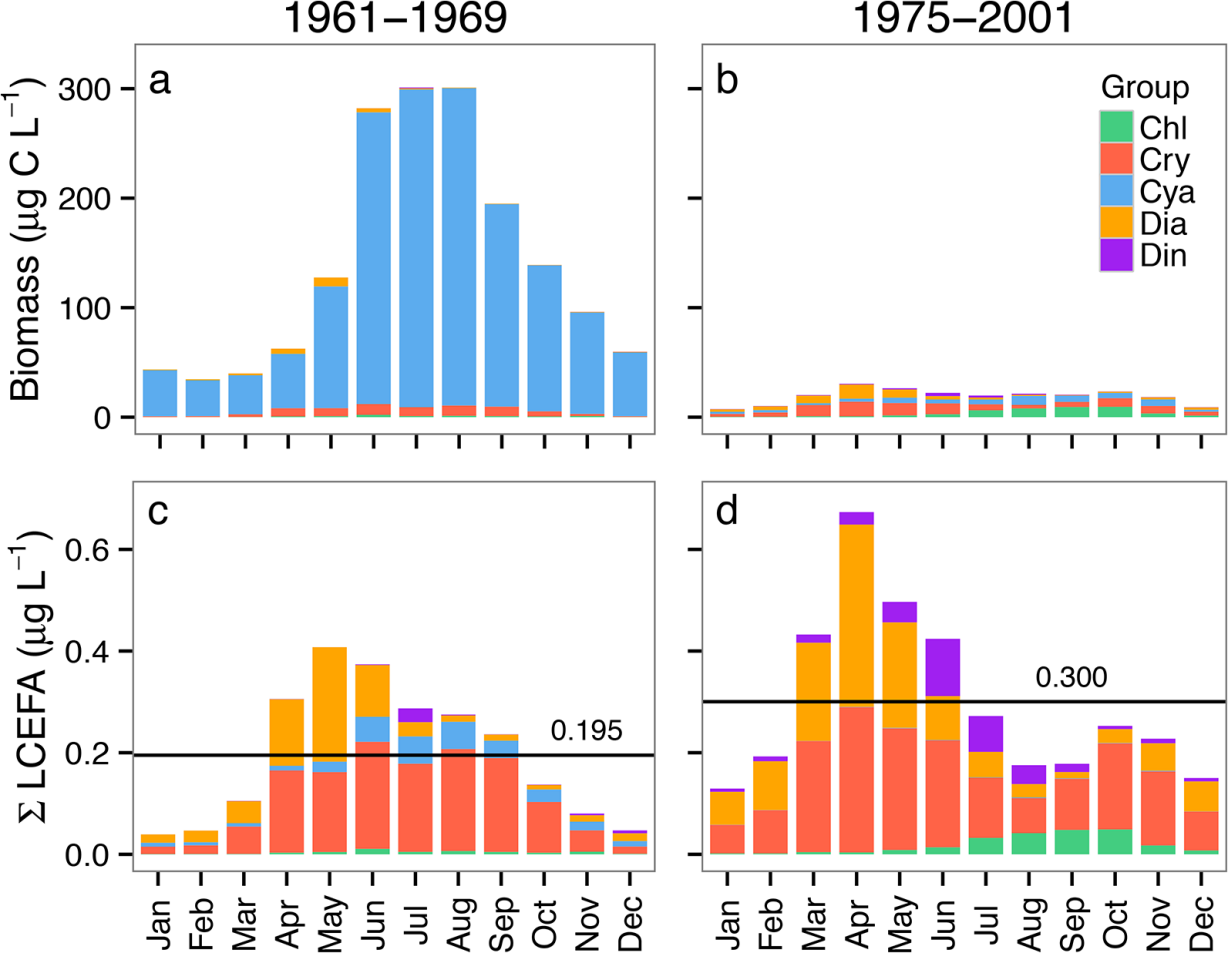
Stacked barplots of observed average monthly phytoplankton biomass (a, b) and calculated LCEFA concentrations (c, d) in Lake Washington. Panels (a) and (c) show the group composition during the eutrophic years (1961–1969), and panels (b) and (d) summarize the oligotrophic years (1975–2001) for biomass and LCEFA, respectively. Group specific LCEFA are calculated following Eq 2. The mean calculated annual LCEFA value across years within each time period is superimposed as a line on panels c and d. Abbreviations follow Fig 1.

**Fig 7:**
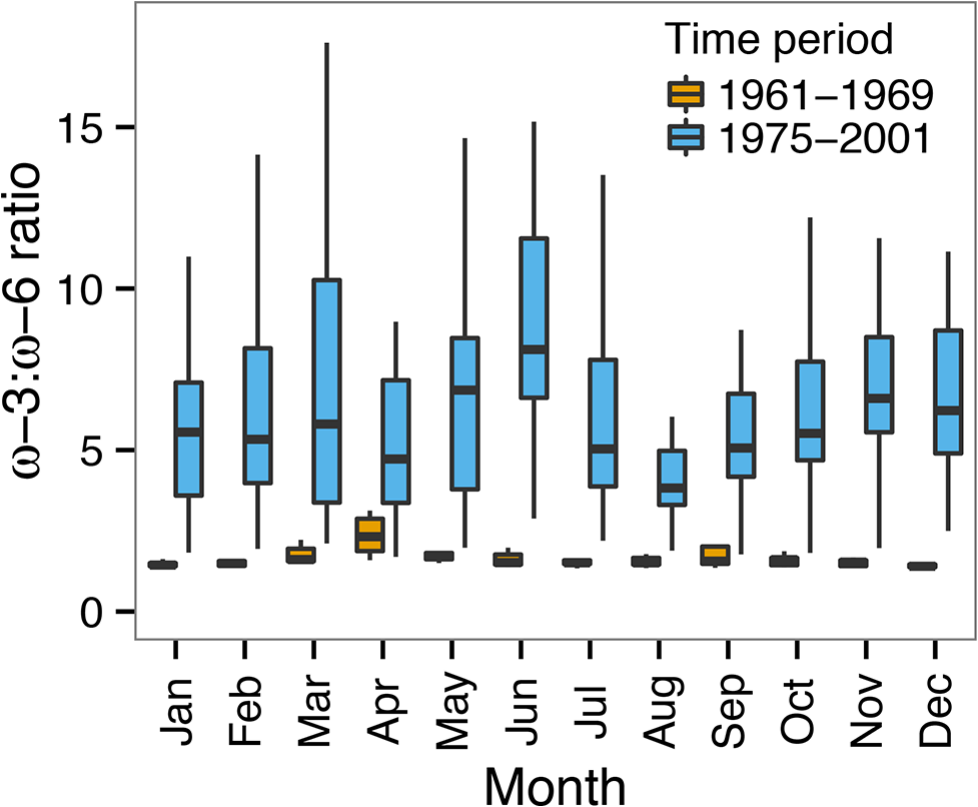
Lake Washington monthly ɷ-3:ɷ-6 fatty acid ratios calculated from phytoplankton biomass from 1961–2001. The boxplot colors represent the time periods and boxplot dimensions follow description in Fig 4. The y-axis is set to show the extent of whiskers, excluding extreme outliers with ratio values >18.

Visualizations of the Lake Washington phytoplankton biomass (Fig 5), and calculated LCEFA (Fig 6) and ɷ-3:ɷ-3 ratios (Fig 7) show the relative seasonal dynamics of LCEFA concentrations attributable to phytoplankton in this system. Algal biomass was dominated by cyanobacteria in the eutrophic phase (1961–1969; Figs 5A and 6A) and was more taxonomically diverse during the later clear-water phase (1975–2001; Figs 5A and 6B). The seasonal timing of maximal biomass was 2–4 months later in the eutrophic years relative to the clear-water phase (Fig 5B and 5C), and overall monthly median phytoplankton biomass was much higher in the eutrophic years (~ 30–350 μg C L^-1^) compared with the clear-water years (~10–40 μg C L^-1^). The analyses of calculated LCEFA show that despite major differences in community biomass and peak biomass timing between these phases, diatoms and cryptophytes are the primary drivers of LCEFA attributable to algae in both phases (Fig 6C and 6D). The average total annual estimated algal-derived LCEFA was 35% higher during the clear-water phase (0.300 μg LCEFA L^-1^) relative to the eutrophic phase (0.195 μg LCEFA L^-1^), and the timing of maximal LCEFA content was ca. 1 month earlier during the clear-water phase (April) compared to the eutrophic phase (May; Fig 6C and 6D).

The calculated monthly phytoplankton-derived ɷ-3:ɷ-6 fatty acid ratios for all observations in both time periods are shown in Fig 7. The ɷ-3:ɷ-6 ratios were variable across years with a median ratio greater than five for all but two months (April and August) and greater than three for all months during the clear-water time period. In contrast, the ɷ-3:ɷ-6 ratios were never greater than 3 during the eutrophic years and were generally consistently low across years.

## Discussion

### Quantifying drivers of algal fatty acids

Our approach, which synthesizes results from many published studies of phytoplankton fatty acids, offers a novel evaluation of the relative importance of ‘nature’ (phylogeny) or ‘nurture’ (environment) for algal fatty acids at an unprecedented scale, encompassing many permutations of culture conditions for many variables and more than 200 unique species. Our results clarify that the primary underlying mechanism determining fatty acid contents in aquatic habitats in nature is the phytoplankton community composition. Thus, changes to environmental conditions in aquatic ecosystems are likely to drive basal food quality in aquatic ecosystems indirectly through changes in phytoplankton community composition due to stratification, algal competition, or trophic pressures [57] rather than directly, by altering synthesis of essential fatty acids. Phylogenetic relationships are known to be a major driver of fatty acid patterns in primary producer groups both in the lab or as seston in the field [8,13,44–50,70]. However, the relative importance of phylogeny and environmental conditions on algal fatty acids and food quality has remained opaque. This is because studies assessing the importance of phylogeny on diverse algae cultures generally control for, or do not account for, environmental conditions, whereas studies assessing effects of environmental conditions on algal fatty acids generally only manipulate one or two conditions for a few algal species at a time. Recently, researchers have started addressing this knowledge gap by concurrently manipulating culture conditions for representative genera from different major groups [52,53,71], but only several often-studied ‘model’ phytoplankton species have been evaluated in this way.

Taxonomic group explained the greatest proportion of the total variation in fatty acid profiles in this study (~36–44%), but was lower than what was reported for marine phytoplankton (e.g., 61% [8]), freshwater phytoplankton (e.g., 66% [45]), and similar to what was found in a survey of marine macrophyte phyla (e.g., 36–41% [70]). Plausible explanations for the decreased variation explained in this dataset relative to other analyses are that our synthesis covers a much larger number of taxa, uses less fatty acids, includes studies data from highly variable culture conditions, and also includes ‘outlier’ fatty acid profiles. Nonetheless, clear algal-group level separation was evident in the multivariate visualization of the fatty acid patterns (PCA; S1 Fig), and is very similar to previous analogous visualization analyses [8,44,45,70,72]. To our knowledge, Dalsgaard et al. [8] were the first researchers to quantify the explanatory power of algal group affiliation on multivariate fatty acid signatures, using a partial least squares regression approach. However, all related research that we are aware of has generally been based on much smaller datasets segregated by freshwater and marine algae, and has not comparatively quantified both group affiliation and culture conditions on algal fatty acids.

Our results also corroborate previous research on individual algal strains for all major phytoplankton groups, confirming that light, temperature, nutrient status, and salinity do clearly affect phytoplankton fatty acids (e.g., DISTLM marginal tests, S3 Table). Extensive research has shown that fatty acids within particular algal groups respond significantly to manipulations of environmental conditions, both in the lab [51,52] and in the field (e.g., [73,74]), so this is not unexpected. For example, temperature [51,71], light [52,75], nutrients [53,76] CO_2_ [77], and growth phase [71,78] are all well known to affect algal total lipids and fatty acid profiles. Marine and freshwater habitats are generally expected to continue to experience increases in surface water temperatures [14,15]. In estuaries, changes in the rates of freshwater inflow and increased temperatures can strengthen thermal stratification and alter salinity regimes [79]. It is therefore reasonable that plankton ecologists may expect that the net synthesis of EFA by algae would be subjected to environmental perturbations, such as coastal eutrophication or climate change.

We evaluated the relationships between the top-ranked environmental variables for each fatty acid dataset identified from the DISTLM analysis. After group, salinity of the culture conditions is a particularly important factor in driving fatty acid composition and total LCEFA (Figs 1–3), although, it explained only an additional 2–3% of the remaining variation in each dataset (Table 1). Correlation tests between salinity and % LCEFA of each species by algal group (Fig 3) showed that higher salinity chlorophytes and cryptophytes had relatively higher % LCEFA than the freshwater strains, and cyanobacteria have uniformly low LCEFA regardless of salinity. Diatoms appear to have a widely variable but generally unimodal relationship with relatively high % LCEFA at high salinities, and particularly high % LCEFA content (e.g., 25–45%) at intermediate salinity of ~18 ppt. While the linear correlations with salinity were not significant for diatoms or haptophytes, the results show a general trend of larger range and higher % LCEFA maxima for higher salinity profiles in all groups except cyanobacteria. Total LCEFA in the % DW dataset was also positively correlated with salinity (Fig 2D). The higher LCEFA content in marine relative to freshwater phytoplankton is consistent with a smaller analysis of published values in Brett *et al*. [38], suggesting that these differences are not just from an overabundance of marine profiles or a bias from aquaculture related studies, which often maximize study efforts LCEFA-rich phytoplankton taxa [38]. These results suggest that algal communities in saltwater systems may have higher potential for generating LCEFA than freshwater or very low salinity counterparts, particularly in communities rich in chlorophytes, cryptophytes, haptophytes, and diatoms. Increased LCEFA of phytoplankton at the base of the food web in saltwater habitats is one potential explanation for increased fisheries yield [80] or for higher trophic efficiency [39] observed in marine relative to freshwater ecosystems.

Culture conditions explained less total variation in the % FA dataset than in the FA % DW dataset (Table 1). Across all algal strains covered in our meta-analysis, we found a weak but general negative relationship between light intensity and total algal LCEFA % DW (Fig 2B). This result is consistent with theory, developed from research on individual algal taxa (e.g., *Nannocholoropsis*), that PUFA-rich galactolipids (particularly high in EPA and ARA) accumulate at lower irradiances as a result of increased growth of chloroplasts and thylakoid membranes for higher photosynthetic capacity [81,82]. The total hours of light variable accounted for the second most variation (11%) of any explanatory variable in the FA % DW dataset (Table 1), and there was a stronger relationship between total h of light d^-1^ and total LCEFA % DW. Taken together, these novel findings imply that for diverse algal groups, relatively shorter light duration (e.g., ~12 h d^-1^) at the lower end of intermediate light intensities where light saturation is achieved (e.g., ~80 μmol m^-2^ s^-1^), might be ideal for maximizing LCEFA content in algal cultures. Interestingly, temperature explained only little variation in the full fatty acid profiles in each dataset (Table 1). Algal polyunsaturated fatty acid content generally is generally known to have an inverse relationship with temperature [51,83,84], but the LCEFA summary category was not negatively correlated with temperature (Fig 2C), and this lack of relationship did not change when the profiles cultured at the rather extreme low temperatures of 4°C were removed. LCEFA content was highest at the ‘intermediate’ temperature of ~25°C, which is also consistent with results reported in Renaud *et al*. [83].

Changes to multivariate fatty acid profiles may be complex, and therefore difficult to summarize, because general patterns may be driven entirely by certain individual or classes of fatty acids. For example, Piepho *et al*. [52] showed that for several algal taxa under both nutrient limitation and replete conditions the concentration of total fatty acids, SAFA, and MUFA all increased with increased light intensity; however, PUFA content was not significantly affected by light intensity in their experiments. In addition, comparisons between the results of different studies can be confounded by differences between studies in the use of either compositional data or concentration data. Culture conditions were less important in the % FA dataset than in the FA % DW dataset. It is currently unknown whether this difference is due to the increased sample size in the % FA data, which included ca. 6 times as many fatty acid profiles as the FA % DW dataset. It is thus not possible to determine with our analysis whether this apparent difference between the two metrics is a result of a real difference in the sensitivity of algal fatty acids to culture conditions.

Given the large variation in culture methodologies and diversity of the original goals of the primary research we compiled, perhaps it is not surprising that there is a large amount of total variation left unexplained in our analyses. The multivariate models described 48.4% of the total variation in algae fatty acid signatures for the % FA, and 56.8% for the FA % DW datasets (Table 1). There are several potential reasons for this unexplained variation. First, this synthesis approach could not account for all details in culturing conditions used by researchers. For example, while we documented culture media used (see S1 Dataset), many researchers reported imprecise names or modifications of standard culture media. Culture flask types and volumes were also not always reported; we attempted to control for this by excluding experiments where algae were cultured in very large vats (see S1 File). Second, we did not screen away ‘outlier’ samples from this analysis. For example, the original research may have been designed to culture algae under fairly extreme light and temperature conditions. All profiles that met our *a priori* criteria (see discussion in S1 File) were included in this analysis. Third, several of the fatty acid variables were calculated summary categories (e.g., MUFA, other PUFA) causing loss of some information. Finally, we did not attempt to quantify or estimate fatty acid data measurement error in the original papers. Despite these uncertainties, the key conclusion obtained from each dataset is that the high order taxonomic group is the primary driver of algal fatty acids.

### Implications to aquatic food webs

Our results show both the extent of variation and overall group hierarchy of LCEFA content among the different algal groups (Fig 4). The medians and 25^th^-75^th^ percentile boxes showed that there are generally 3 categories of LCEFA content: high (dinoflagellates, diatoms, and cryptophytes); medium (haptophytes and chlorophytes); and low (cyanobacteria). This hierarchy follows the food quality rankings, for a subset of these algal groups to freshwater zooplankton, described by Brett & Müller-Navarra [39], based on EFA, and Park *et al*. [62], based on the carbon:phosphorous ratio of these resources. Our meta-analysis extends the relative rankings reported previously by also summarizing the LCEFA content of dinoflagellates and marine haptophytes, by reporting ɷ-3 and ɷ-6 content, and greatly increasing the sample size for each algal group (Table 2). The use of relative LCEFA alone as an index of food quality is limited because this simply metric cannot account for the fact that certain taxa, particularly dinoflagellates, which have relatively high DHA content, may also be toxin-producing and poor quality food for consumers [85]. Despite this consideration, total LCEFA content synthesized by phytoplankton can still be used to estimate LCEFA produced by phytoplankton from existing community time-series where no lipids were preserved.

We used the calculated average LCEFA content of the algal groups from our meta-analysis to identify the relative phytoplankton-derived group-specific contribution of LCEFA across several decades at seasonal scales in a large, urbanized, and heavily perturbed freshwater ecosystem. The results show that Cyanobacteria, particularly *Oscillatoria*, were a large contributor to total annual average phytoplankton biomass in Lake Washington until the early 1970s, but were generally rare after 1972 (Fig 5A), as a consequence of cessation of the dumping of raw sewage into the system from 1942–1962 (reviewed in [65,67]). Concurrent with the decline in *Oscillatoria*, the abundance of diatoms and the cladoceran generalist consumer *Daphnia* started to increase in 1973, with substantial and sustained population increases in 1977 [66]. Seasonal calculations for both the eutrophic and clear-water phases demonstrate that diatoms and cryptophytes are a critical source of LCEFA in this system, even when they are relatively rare (Fig 6). In addition, cryptophytes appear to be a relatively consistent source of algal LCEFA in this system. It is informative to consider these baseline LCEFA fluctuations in Lake Washington in the context of *Daphnia* abundance; for instance, in 1977–78, two early years of greatest initial increase of *Daphnia* in Lake Washington [66], the diatom biomass and calculated LCEFA content was also much lower than nearby years. Thus, we acknowledge that these LCEFA estimates, which are related to the phytoplankton abundance, do not account for LCEFA bound up in diverse grazers including heterotrophic protists or zooplankton.

Long-term LCEFA dynamics we show here should be interpreted with caution and treated as hypotheses. These hypotheses offer a few interesting perspectives and potential interpretations of the Lake Washington data. Our analyses indicate that optimal ɷ-3:ɷ-6 fatty acid ratios for *Daphnia* (≥3–5) [69] were rarely if ever achieved in Lake Washington during the period of cyanobacteria domination. April was the only month during this time period where these ratios were almost achieved. Our analysis indicates that these ratios were likely commonly exceeded during the clear-water phase (Fig 7), with maximal peaks in ɷ-3:ɷ-6 ratios shifting one month earlier from the eutrophic period, to March. Maximal estimated LCEFA content also occurs ca. one month earlier in the year during the clear-water phase, likely as a result of earlier [63] and larger spring blooms of diatoms in this latter phase (Fig 6). Our estimates of the timing of peak LCEFA concentrations in Lake Washington during the clear-water phase (April) corresponded closely to peak concentrations of the same LCEFA reported in Ravet *et al*. [43] measured ~9 years after the end of our time series.

The application of the algal group specific total LCEFA content at the ecosystem scale to existing phytoplankton time-series has important caveats and limitations. We focused our calculations on only 3 LCEFA; these calculations therefore do not account for several C_18_ ɷ-3 and ɷ-6 fatty acids such as 18:2ɷ-6 (LIN) or 18:3ɷ-3 (ALA) that are also often considered as essential fatty acids for consumers [10]. While some consumers can convert these C_18_ EFA to LCEFA, primary consumers are generally not efficient at these conversions and this trophic modification varies substantially among taxa and depending upon conditions. Thus, our analysis is conservative in the estimation of total algal-derived EFA. The choice of these three conditionally essential LCEFA for broad analyses is not unprecedented [86,87]. Importantly, the strictly phytoplankton based estimates used here do not calculate total ecosystem seston LCEFA, which may also be stored in heterotophic protists [46,55,88]. It is well established that heterotrophic protists play key roles in aquatic food webs by trafficking and repackaging picoplankton production into metazoan consumers [89], acting as ‘trophic upgraders’ [90,91] by helping to synthesize essential nutrients for zooplankton consumers. For example, in nature, lake seston EPA content may be largely explained by the abundance of ciliates [55]. Ciliates may synthesize LCEFA *de novo* [92], or at the very least, build C_20_ ɷ-3 from precursor C_18_ ɷ-3 fatty acids (reviewed in [88]). Our approach could not account for ciliates, which may help explain why our estimates of algal-derived LCEFA content at the ecosystem scale are lower than concentrations of LCEFA measured from unsorted seston samples in other systems [48,55].

### Conclusions

There is large variation in the quality of different phytoplankton groups as food for consumers; thus it is important to consider that not all phytoplankton production is “equal” [85]. The three key factors that define the ‘quality’ of a dietary resource to a given consumer are the physical attributes affecting ingestibility, the content of essential nutrients, and resource toxicity [39,56]. The most important of these factors affecting food quality at the ecosystem scale is arguably the availability of essential biomolecules, including amino acids, fatty acids, sterols, because heterotrophs cannot synthesize these required resources at sufficient levels or at all. Essential fatty acids are one class of several important biochemical determinants of food quality [56,93]. Experiments with aquatic model consumer taxa have demonstrated the importance of co-limitation by multiple essential nutrients in addition to EFA, including sterols and amino acids [91,94,95]. Our approach, which summarizes a general ranking of phytoplankton food quality based on fatty acids, is not meant to discount other factors or biochemical constituents of phytoplankton that may regulate basal heterotrophs, but provides a baseline perspective of the relative quality that is based on extensive previous research.

Why focus on lipids generally and specifically long chain essential fatty acid dynamics as a proxy for food quality? Researchers are increasingly recognizing the broad and critical role that lipids play in structuring aquatic ecosystems [40]. Total lipid content of fish is established as a useful predictor for future egg production [96], and LCEFA appear to be a particularly important component of lipids for eggs [86,87]. The approach we used to calculate environmental LCEFA, based on published phytoplankton data, can thus be complimented by traditional approaches involving direct measurements [49], and modeling of environmental LCEFA monitoring based on satellite imagery of ocean color [21]. The case study of LCEFA dynamics in Lake Washington is broadly relevant to other systems, as shifts towards cyanobacteria dominated communities in lakes [97], and marine ecosystems [98], are expected in the future, and such changes will certainly have consequences to total LCEFA content and ɷ-3:ɷ-6 fatty acid ratios in diverse aquatic ecosystems. For example, an analysis of a 35-year marine time series has shown that cyanobacteria blooms are occurring earlier in the year and the magnitude of blooms have increased interannual variability in the Baltic Sea [99]. Interestingly, in Lake Washington, Francis *et al*. [66] showed that general plankton community stability was lowest during periods dominated by cyanobacteria; this raises the question of whether LCEFA content or ɷ-3:ɷ-6 fatty acid ratios may be predictors of stability in aquatic communities.

This analysis has shown that while environmental conditions affect the fatty acid composition in phytoplankton at the species level, changes in these conditions, as a result of climate change, eutrophication, or other affects of water management, will not likely be the direct cause of wholesale changes in essential fatty acids in aquatic ecosystems. Phytoplankton fatty acids, and by extension, the food quality of phytoplankton communities in nature, are therefore likely to be determined primarily by the taxonomic composition of the community [57,85]. We have demonstrated a novel approach for using phytoplankton group LCEFA content to estimate dynamics of LCEFA and ɷ-3:ɷ-6 fatty acid ratios for long-since collected community samples. The average LCEFA % DW dataset calculated from this meta-analysis could be used in conjunction with any phytoplankton community dataset to calculate similar estimates of LCEFA availability in real-world seston samples with moderately high-resolution phytoplankton data.

## Supporting Information

S1 Dataset A .csv file of all compiled FA profiles. (CSV)

S1 File Supplementary Methods. Detailed description of the criteria used for dataset inclusion in the literature search, the different kinds of data (e.g., composition or concentration based), detection limits, and the environmental variables in each of the published studies.
(PDF)

S1 Fig Principal components analysis (PCA) of 11 fatty acids of phytoplankton profiles (% FA dataset). (a) The PCA includes the six dominant phytoplankton groups in all culture conditions (n = 666 profiles; abbreviations follow Fig 1). Fatty acid data were arcsine-square root transformed; PCs 1 and 2 (pictured) accounted for 51% of the total variation and PC3 for 15%. (b) Plot of correlations between the fatty acid variables and PCA scores, where arrow length identifies the Pearson correlation for each variable to PC1 and PC2.
(TIF)

S2 Fig Barplot of the relative food quality indices (FQI) calculated for both types of fatty acid data. The FQI is based on Σ LCEFA, as described in Eq 1. All raw fatty acid profiles (n = 666 for % FA and n = 101 for FA % DW), under all culture conditions, within these six algal groups (abbreviations follow Fig 1) were first averaged to 208 and 55 total unique species in each dataset, respectively (see Methods). The numbers of unique species averages in each index are plotted above each bar.
(TIF)

S1 Table Summary of all literature sources used in this synthesis. A total of 58 studies of the 399 screened had sufficient data on the culture conditions (light intensity, light duration, temperature, nutrients, growth phase, and salinity) and consistent FA variables for analysis beyond initial screening (see S1 File).
(PDF)

S2 Table Number of FA profiles in each of the algal groups in the full meta-analysis data set. Table is organized by FA data type and indicating the six major algal groups that are the focus of analyses. The FA observation (n profiles) in the ‘included’ category is the number of raw profiles that were used in the PCA (S1 Fig) or eligible for inclusion in the DISTLM analysis.
(PDF)

S3 Table Results of DISTLM marginal tests. Results of DISTLM marginal tests, quantifying the relative contribution of algal group affiliation and culture condition variables (following abbreviations of Table 1) for both fatty acid datasets (% FA and FA % DW). The marginal tests result reports the proportion of the variation (prop. var.) explained by each variable, independent of any others (see Methods).
(PDF)
