# Supplementary material for: Partitioning the Relative Importance of Phylogeny and Environmental Conditions on Phytoplankton Fatty Acids
Source: PLoS One. 2015 Jun 15;10(6):e0130053. doi: 10.1371/journal.pone.0130053 (PMC4468072; doi:10.1371/journal.pone.0130053)
Supplement: S1 File — Detailed description of the criteria used for dataset inclusion in the literature search, the different kinds of data (e.g., composition or concentration based), detection limits, and the environmental variables in each of the published studies. (PDF) [file pone.0130053.s002.pdf]

S1 File. Supplementary Methods. Detailed description of the criteria used for dataset inclusion in the literature search, the different kinds of data (e.g., composition or concentration based), detection limits, and the environmental variables in each of the published studies.

1. Dataset inclusion

Using ISI Web of Science searches including the terms 'fatty acids', 'phytoplankton' and each of the phytoplankton group names, we sought out papers that cultured algae in diverse and yet controlled conditions. We also identified additional potential datasets using a combination of prior knowledge of the literature and through tracing the citations of other key studies in the field. We purposely included studies that we expected would produce 'outlier' fatty acid (FA) profiles based on experimental manipulation of conditions expected to affect FA composition. The justification for this varied approach is that phytoplankton FA profiles are reported throughout a diverse literature with very different original study goals.

Relevant studies are from a wide range of disciplines, from research on prospecting for potential sources for energy and natural products, to studies in aquaculture, experimental food webs in aquatic ecology, and general phycological surveys. This made it difficult to make the meta-analysis paper search simple and purely objective and repeatable. Our use of prior knowledge in searching for papers does potentially introduce bias; however, given the unprecedented sample sizes used in this analysis (1145 FA profiles) and the focus particularly on including papers that would manipulated environmental variables, we argue that our conclusions are robust to this sampling bias. We included data from 58 studies out of the 399 studies initially deemed promising using our search criteria (see S1 Table). Below we provide a detailed account of the reasoning behind dataset inclusion, with citations of one or two studies providing

representative examples of these criteria. Exclusion from this meta-analysis does not imply flaws in the original papers, just that they did not meet our particular criteria.

We did not include FA profiles of mixed phytoplankton taxa [e.g., 1], studies that cultured phytoplankton in outdoor holding tanks or culture tanks larger than an arbitrary volume of 100 L volume [e.g., 2] in an attempt to partially standardize potentially important but unknown conditions from such circumstances that make such studies harder to compare with the majority of the studies. For studies with a combination of profiles from different or unknown categories, we used the subset of FA profiles that met the criteria or where key conditions were known or reported by the authors [e.g., 3,4]. We included FA profiles from cultures that were described by the authors as not 'axenic', because this ideal standard is almost never met in culture experiments.

Researchers do not always report the same FA for various reasons. To include data from as many studies as possible, we initially recalculated each published FA profile to comprise of 11 consistent individual FA or categories. The 8 individual ω-3 and ω-6 EFA are: 18:2ω6 (LIN), 18:3ω6 (GLA), 18:3ω3 (ALA), 18:4ω3 (SDA), 18:5ω3, 20:4ω6 (ARA), 20:5ω3 (EPA), and 22:6ω3 (DHA). In addition, we compiled 3 summary fatty acid categories for each profile, including; sum of saturated fatty acids (SAFA), sum of monounsaturated fatty acids (MUFA), and sum of non-EFA PUFA, or 'other PUFA'. The other PUFA variable was calculated by subtracting the total of the 8 individual EFA from the total remaining PUFA in the original datasets. This ensured that the analytical dataset did not have double sampled variables. The other PUFA variable mostly captures the sum of C_{16} PUFA and other relatively rare >C_{18} PUFA.

We did not include FA data from studies that reported a large proportion (e.g., mean across samples >5%) of 'other' or 'unknown' FA [5,6] because we could not assign these other

FA to matching variables in our database. In addition, we included data only from studies that reported data from total FA (e.g., not split by lipid classes), to maintain consistency among the analytical units. We did not include data from studies that reported only the number of carbons and double bonds, without reporting the position of the position of the final double bond from the methyl group [e.g., 7,8] because we could not verify that FA reported this way corresponded to those in our database. Studies that combined key PUFA variables as one were also excluded [e.g., 9] for the same reason.

We did not include studies that did not report information for a majority of the explanatory variables we used in the analysis [10] or studies that did not report the FA data used for their analysis as tables or supplementary material [e.g., 11]. If authors reported multiple extractions from the same algal strain [12,13] or if it was not clear from the methods that experimental conditions varied between these replicates [14], we entered the mean of these values for each distinct strain. We did not remove what we perceived to be potential 'outlier' data from the master file except for in the case of Viso and Marty [13]; concentration data reported in this paper were ca. one order of magnitude lower from other published concentration data for related phytoplankton taxa. In this case, the Viso and Marty [13] proportional data were still used but the concentration data were removed prior to analyses. Finally, in the few cases where our explanatory data is different than what is reported in original papers, or if it was not reported in the original paper, the discrepancy is because these data were provided by the original authors via email correspondence.

2. FA concentrations and composition

Defining a common metric for expressing FA concentration is challenging due to the diverse ways that researchers present data. The lack of a common standard is due to the varied goals of researchers [discussed in 15,16]. Our data compilation effort included on both compositional FA data and several of the most common concentration metrics [FA μg mg^{-1} C, FA % dry weight (DW), and pg FA $cell^{-1}$]. Of the concentration metrics, we focused our statistical analysis on the FA % of DW (hereafter referred to as FA % DW) dataset because this was the only weight-based category with ample within-group taxonomic replication for statistical evaluation of the effect of all variables.

Composition data (% FA of total lipids, hereafter referred to as % FA) was either taken directly from the original paper or calculated from the raw concentration data if that is how the researcher originally presented it. Concentration data was either taken directly from the original paper or calculated by multiplying the given raw proportion data for each FA by the total FA data provided by the authors (FA μg mg^{-1} C, FA % DW, and pg FA $cell^{-1}$) for each sample. Because original FA % DW and pg $cell^{-1}$ data may be reported by the authors as from either the total lipid or total FA (or both), we used only the most commonly reported approach for each of these metrics, which was pg FA $cell^{-1}$ and FA % DW of total lipid content. Concentration data was not included if this distinction was unclear from the original methods, or if the data were presented in alternative formats [e.g., % organic weight (OW), nmol FA $cell^{-1}$, μg x 10^6 $cell^{-1}$, μm^{-3}, mg L^{-1} day^{-1}].

Researchers may report concentration data as μg FA mg $carbon^{-1}$, particulate organic carbon, or 'dry biomass'. We initially compiled the raw data for each of these types of data (see the S1 Dataset), but performed analyses on only the composition data (% FA), FA % DW of total lipid, and the FA μg mg^{-1} C datasets. Our approach provided a relatively simple and consistent

way of maximizing the number of profiles that were reported in a common way. These decisions were made prior to analyses.

3. Detection limits

Detection limits varies among papers, but most studies will report FA compositional data for all FA that comprise >0.05% of the total FA. Below this limit it is common for authors to indicate that samples have 'trace' amounts of particular FA because non-quantified peaks appear on chromatograms. When authors report 'trace' we entered a non-zero value in the raw file defined as the number just below the value defined by the authors for trace in their data. For example, Dunstan et al. [17] report that 'trace' is <0.05%; in these cases we entered as 0.04% in our file. When original study authors did not report trace values, we assumed that FA in these samples was below detection limits and cannot be differentiated from zero.

If original authors did not report certain very commonly identified PUFA that are in virtually all FA standards (e.g., ALA, ARA, DHA), we assumed that this omission was because these FA were not present above detection limits, and we therefore treat these as zeros in the database. The reasoning for this is that many algal strains do not have these FA that are common in the majority of studies, and dropping these profiles from later analyses because of 'missing data' would unfairly penalize these strains or studies that only worked with these strains. There is one PUFA we included in our FA list, which is not commonly in standards and is usually only identified in studies working with dinoflaggelates (18:5ω3). We entered 'nr' (not reported) in the master database for this FA when it was not reported. However, following the same logic used above 'nr' entries for this FA were assumed to indicate the absence of this FA above detection limits in the sample, and these values were therefore treated as zeros in our analyses.

4. Variables

The growth condition explanatory variables reported in nearly all studies were entered as described by the original papers. In some cases, certain explanatory variables (light, culture technique) was obtained from methods sections of other papers by the same author or other papers that the primary paper cited for general methodological details [e.g., 18] or by direct email communication with the authors. The following variables (in numbered sub-headings below) were the variables included in distance based linear model [DISTLM; 19] analysis (See the Analysis section below and in the main text). The raw dataset also has other meta-data consistently reported by the original authors, including additional treatment explanations, growth media used, and culture container used. These various other categories were not reported consistently enough to be formally included in the analyses (see the S1 Dataset).

4.1. Group (high order taxonomic group; categorical)

For each taxon FA profile entered into the database (e.g., each unique row) the original genus and species names reported by the authors was cross-referenced with AlgaeBase [20] to ensure correct and consistent spelling and align current taxonomic affiliations for the taxonomic designations of Phylum, Class, and Order, Genus and Species. The operative (i.e., analytical) group unit was later defined by collapsing and splitting some of these categories; for example we categorized multiple Classes commonly known as 'diatoms' as one distinct group rather than lumping these classes with Eustigmatophyceae or Chrysophyceae, which are all also presently aligned with Ochrophyta [20].

We therefore categorized the raw data into the following 9 groups: Diatoms (Fragilariophyceae, Bacillariophyceae, Coscinodiscophyceae); Dinoflagellates; Chlorophytes; Cyanobacteria; Ochrophyta (i.e., non-diatom Classes: Chrysophyceae, Raphidophyceae, Eustigmatophyceae, Xanthophyceae); Cryptophytes; Euglenoids (Euglenozoa); Rhodophyta; and Haptophyta. Due to limited sample sizes in the Ochrophyta, Euglenoids, Rhodophyta, our analyses did not include these groups (see Analysis section below).

4.2. Light (intensity; continuous)

This variable is reported in the literature as W m^{-2}, μ Mol m^{-2} s^{-1}, or μE m^{-2} s^{-1}, or in simply “cool fluorescent light”, or Lux. We assumed the following conversions to express all values as μ Mol m^{-2} s^{-1} [21]:

1 μ Mol m^{-2} s^{-1} = 1 μ E m^{-2} s^{-1}

1 μ Mol m^{-2} s^{-1} = 0.218 W m^{-2}

1 μ Mol m^{-2} s^{-1} = 74 Lux (for cool white fluorescent bulb)

1 μ Mol m^{-2} s^{-1} = 33 Lux (for plant growth fluorescent bulb)

If bulb type was not reported, we used the mean of the conversion factor for the two fluorescent bulb types. If a range of light values was reported [e.g., 12], we used the mean of these values. If conditions were not reported, data were obtained from personal communication with the authors [e.g., 22].

4.3. Light2 (hours light; continuous)

Entered as the number of hours within the 24 hour day that the authors reported having the lights on in the growth chambers.

4.4 Temp (temperature; continuous)

This is the temperature (°C) that is reported for each algal experiment or treatment (if temperature was a treatment). If a range of values was reported we used the mean of the range.

4.5. Salinity [salinity in parts per thousand (ppt), equivalent to gL^{-1}; continuous]

For experiments with salt-water taxa, the salinity is entered as reported by the authors, when they report it. If the original authors were clearly culturing algae in saltwater but did not report the salinity we assigned an arbitrary value of 32 ppt to these samples. All profiles for freshwater experiments were assigned a value of 0.

4.6. Nutrient status (binary proxy for diverse conditions; categorical)

Previous research has shown that FA profiles in microalgae are sensitive to nutrient levels [16]. Diverse experiments have manipulated many potentially limiting nutrients (N, P, Fe), and the degree of nutrient limitation among experiments varies greatly depending on the author's original question. For example, nutrient limitation may be investigated as a binary, low-high value [15] or along a gradient of minor to severe limitation [16]. In addition, experiments have also shown that growth phase affects phytoplankton FA [17,23,24].

Here, we categorized these diverse experimental manipulations across all studies into a binary, categorical 'nutrient' indicator factor as either 'limited' or 'replete'. All FA profiles from studies were categorized as 'limited' when the authors either 1) limited concentration of a key nutrient, or 2) manipulated experimental conditions which resulted in the cultures experiencing nutrient limitation, or if extractions were performed when cultures were in a 'stationary' growth

phase [23,e.g., 25]. There is often less information about algal culture conditions in studies where the original objective of algal cultures was to grow biomass for feeding to heterotrophs. In these cases, which did not report algal culture growth phase or nutrient status, we assumed that the cultures were cultured in a nutrient replete status for the sake of producing biomass for the experiments [e.g., 26,27].

5. Analysis

We used a distance-based linear model (DISTLM) to quantify the relative contribution of categorical algal group affiliation and multiple culture condition variables, on multivariate algal FA composition [19]. The analysis was run on two distinct FA datasets [% FA of total lipids (% FA), FA % of dry weight (FA % DW)]; there were 6 taxonomic groups considered in these analyses. We could not perform meaningful or directly comparable DISTLM analyses using the FA μg mg^{-1} C data due to differences in the number of groups represented and due to the low number of taxa profiles within each group (S2 Table).

References

1. Brett MT, Müller-Navarra DC, Ballantyne AP, Ravet JL, Goldman CR. *Daphnia* fatty acid composition reflects that of their diet. Limnol Oceanogr. 2006;51: 2428–2437.
2. Pennarun A-L, Prost C, Haure J, Demaimay M. Comparison of two microalgal diets. 1. Influence on the biochemical and fatty acid compositions of raw oysters (*Crassostrea gigas*). J Agric Food Chem. 2003;51: 2006–2010.
3. Renaud SM, Parry DL, Thinh LV, Kuo C, Padovan A, Sammy N. Effect of light-intensity on the proximate biochemical and fatty acid composition of *Isochrysis* sp and *Nannochloropsis oculata* for use in tropical aquaculture. J Appl Phycol. 1991;3: 43–53. doi:10.1007/bf00003918
4. Chen Y-C. The biomass and total lipid content and composition of twelve species of marine diatoms cultured under various environments. Food Chem. 2012;131: 211–219. doi:10.1016/j.foodchem.2011.08.062

5. Alonso DL, Belarbi EH, Fernandez-Sevilla JM, Rodriguez-Ruiz J, Grima EM. Acyl lipid composition variation related to culture age and nitrogen concentration in continuous culture of the microalga *Phaeodactylum tricornutum*. Phytochemistry. 2000;54: 461–471. doi:10.1016/s0031-9422(00)00084-4

6. Arendt KE, Jónasdóttir SH, Hansen PJ, Gartner S. Effects of dietary fatty acids on the reproductive success of the calanoid copepod *Temora longicornis*. Mar Biol. 2005;146: 513–530. doi:10.1007/s00227-004-1457-9

7. Lourenco SO, Barbarino E, Mancini-Filho J, Schinke KP, Aidar E. Effects of different nitrogen sources on the growth and biochemical profile of 10 marine microalgae in batch culture: an evaluation for aquaculture. Phycologia. 2002;41: 158–168. doi:10.2216/i0031-8884-41-2-158.1

8. Breuer G, Lamers PP, Martens DE, Draaisma RB, Wijffels RH. The impact of nitrogen starvation on the dynamics of triacylglycerol accumulation in nine microalgae strains. Bioresour Technol. 2012;124: 217–226. doi:10.1016/j.biortech.2012.08.003

9. Breteler WCMK, Schogt N, Rampen S. Effect of diatom nutrient limitation on copepod development: role of essential lipids. Mar Ecol Prog Ser. 2005;291: 125–133.

10. Lang IK, Hodac L, Friedl T, Feussner I. Fatty acid profiles and their distribution patterns in microalgae: a comprehensive analysis of more than 2000 strains from the SAG culture collection. BMC Plant Biol. 2011;11: 124. doi:10.1186/1471-2229-11-124

11. Araujo SD, Garcia VMT. Growth and biochemical composition of the diatom *Chaetoceros* cf. wighamii brightwell under different temperature, salinity and carbon dioxide levels. I. Protein, carbohydrates and lipids. Aquaculture. 2005;246: 405–412. doi:10.1016/j.aquaculture.2005.02.051

12. Volkman JK, Jeffrey SW, Nichols PD, Rogers GI, Garland CD. Fatty-acid and lipid-composition of 10 species of microalgae used in mariculture. J Exp Mar Biol Ecol. 1989;128: 219–240.

13. Viso AC, Marty JC. Fatty acids from 28 marine microalgae. Phytochemistry. 1993;34: 1521–1533. doi:10.1016/s0031-9422(00)90839-2

14. Guedes AC, Amaro HM, Barbosa CR, Pereira RD, Malcata FX. Fatty acid composition of several wild microalgae and cyanobacteria, with a focus on eicosapentaenoic, docosahexaenoic and alpha-linolenic acids for eventual dietary uses. Food Res Int. 2011;44: 2721–2729. doi:10.1016/j.foodres.2011.05.020

15. Piepho M, Arts MT, Wacker A. Species-specific variation in fatty acid concentrations of four phytoplankton species: does phosphorus supply influence the effect of light intensity or temperature? J Phycol. 2012;48: 64–73.

16. Bi R, Arndt C, Sommer U. Linking elements to biochemicals: effects of nutrient supply ratios and growth rates on fatty acid composition of phytoplankton species. J Phycol. 2014;50: 117–130.

17. Dunstan GA, Volkman JK, Barret SM, Garland CD. Changes in the lipid composition and maximization of the polyunsaturated fatty acid content of three microalgae grown in mass culture. J Appl Phycol. 1993;5: 71–83.

18. Dunstan GA, Volkman JK, Barrett SM, Leroi JM, Jeffrey SW. Essential polyunsaturated fatty-acids from 14 species of diatom (Bacillariophyceae). Phytochemistry. 1994;35: 155–161.

19. Anderson MJ, Gorley RN, Clarke KR. PERMANOVA+ for PRIMER: guide to software and statistical methods. Plymouth, UK: PRIMER-E Ltd; 2008.

20. Guiry MD, Guiry GM. AlgaeBase. World-wide electronic publication, National University of Ireland, Galway [Internet]. 2014. Available: http://www.algaebase.org

21. Sager JC, Mc Farlane JC. Radiation. In: Langhans RW, Tibbits TW, editors. Plant Growth Chamber Handbook. Ames, Iowa: North Central Regional Research Publication No. 340; 1997. pp. 1–30. Available: http://www.controlledenvironments.org/Growth_Chamber_Handbook/Ch01.pdf

22. Taipale S, Strandberg U, Peltomaa E, Galloway AWE, Ojala A, Brett MT. Fatty acid composition as biomarkers of freshwater microalgae: analysis of 37 strains of microalgae in 22 genera and in seven classes. Aquat Microb Ecol. 2013;71: 165–178. doi:10.3354/ame01671

23. Liang Y, Beardall J, Heraud P. Changes in growth, chlorophyll fluorescence and fatty acid composition with culture age in batch cultures of *Phaeodactylum tricornutum* and *Chaetoceros muelleri* (Bacillariophyceae). Bot Mar. 2006;49: 165–173. doi:10.1515/bot.2006.021

24. Schwenk D, Seppala J, Spilling K, Virkki A, Tamminen T, Oksman-Caldentey KM, et al. Lipid content in 19 brackish and marine microalgae: influence of growth phase, salinity and temperature. Aquat Ecol. 2013;47: 415–424. doi:10.1007/s10452-013-9454-z

25. Ahlgren G, Gustafsson IB, Boberg M. Fatty acid content and chemical composition of fresh-water microalgae. J Phycol. 1992;28: 37–50. doi:10.1111/j.0022-3646.1992.00037.x

26. Broglio E, Jónasdóttir SH, Calbet A, Jakobsen HH, Saiz E. Effect of heterotrophic versus autotrophic food on feeding and reproduction of the calanoid copepod *Acartia tonsa*: relationship with prey fatty acid composition. Aquat Microb Ecol. 2003;31: 267–278.

27. Parrish CC, French VM, Whiticar MJ. Lipid class and fatty acid composition of copepods (*Calanus finmarchicus*, *C. glacialis*, *Pseudocalanus* sp., *Tisbe furcata* and *Nitokra lacustris*) fed various combinations of autotrophic and heterotrophic protists. J Plankton Res. 2012;34: 356–375. doi:10.1093/plankt/fbs003
