# Supplementary figures and images for: Partitioning the Relative Importance of Phylogeny and Environmental Conditions on Phytoplankton Fatty Acids
Source: PLoS One. 2015 Jun 15;10(6):e0130053. doi: 10.1371/journal.pone.0130053 (PMC4468072; doi:10.1371/journal.pone.0130053)

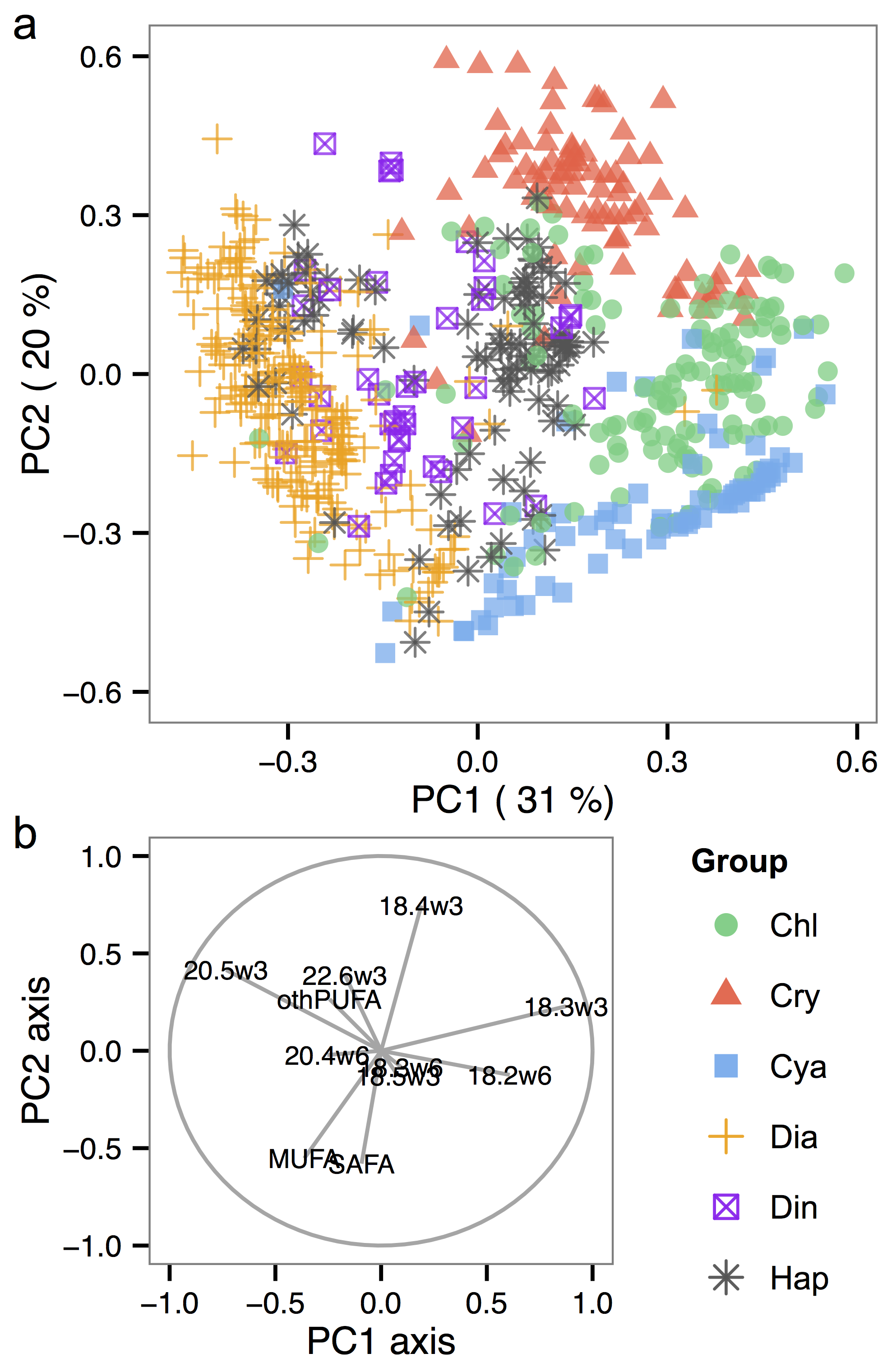

Supplement: S1 Fig — (a) The PCA includes the six dominant phytoplankton groups in all culture conditions (n = 666 profiles; abbreviations follow Fig 1). Fatty acid data were arcsine-square root transformed; PCs 1 and 2 (pictured) accounted for 51% of the total variation and PC3 for 15%. (b) Plot of correlations between the fatty acid variables and PCA scores, where arrow length identifies the Pearson correlation for each variable to PC1 and PC2. (TIF) [file pone.0130053.s003.tif]

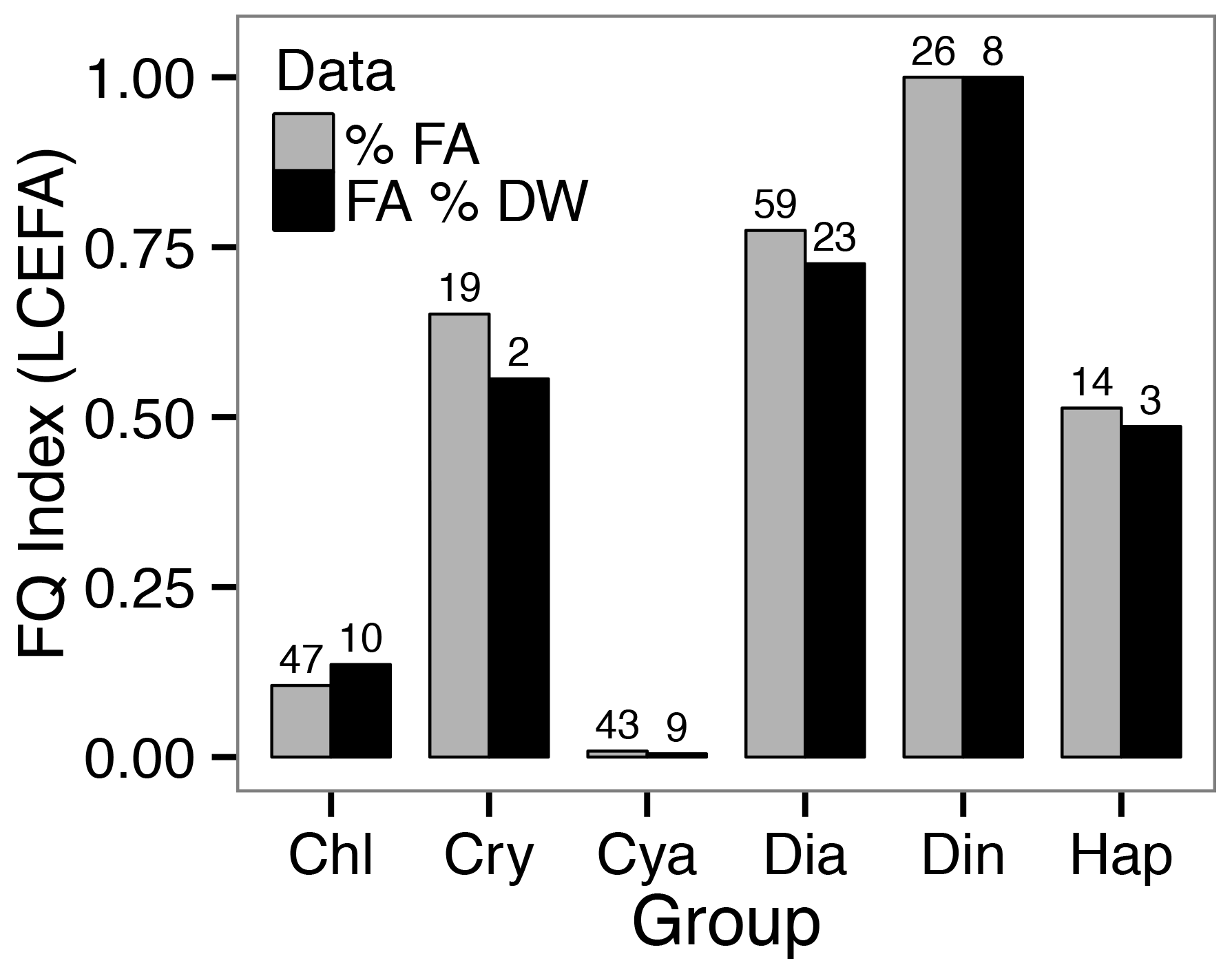

Supplement: S2 Fig — The FQI is based on Σ LCEFA, as described in Eq 1. All raw fatty acid profiles (n = 666 for % FA and n = 101 for FA % DW), under all culture conditions, within these six algal groups (abbreviations follow Fig 1) were first averaged to 208 and 55 total unique species in each dataset, respectively (see Methods). The numbers of unique species averages in each index are plotted above each bar. (TIF) [file pone.0130053.s004.tif]
