# Supplementary material for: Partitioning the Relative Importance of Phylogeny and Environmental Conditions on Phytoplankton Fatty Acids
Source: PLoS One. 2015 Jun 15;10(6):e0130053. doi: 10.1371/journal.pone.0130053 (PMC4468072; doi:10.1371/journal.pone.0130053)
Supplement: S1 Table — A total of 58 studies of the 399 screened had sufficient data on the culture conditions (light intensity, light duration, temperature, nutrients, growth phase, and salinity) and consistent FA variables for analysis beyond initial screening (see S1 File). (PDF) [file pone.0130053.s005.pdf]

S1 Table. Summary of all literature sources used in this synthesis. A total of 58 studies of the 399 screened had sufficient data on the culture conditions (light intensity, light duration, temperature, nutrients, growth phase, and salinity) and consistent FA variables for analysis beyond initial screening (see S1 File Methods).

Source	Groups covered	Study type[1]	Data type[2]	Salinity	n[3] Groups	n[3]~ Taxa	n[3] FA profiles	Treatments, manipulated variables[4]
Ackman & Tocher [1]	Chlorophyta, Chrysophyceae, Diatoms, Dinophyta, Haptophyta, Raphidophyceae, Rhodophyta	Phy	%	Marine	7	11	12	NA
Ahlgren *et al.* [2]	Chlorophyta, Chrysophyceae, Cryptophyta, Cyanobacteria, Dinophyta	Phy	%, %DW	Fresh	5	14	21	growth phase
Bi *et al.* [3]	Cryptophyta, Diatoms, Haptophyta	Phy	%, ug mg C^{-1}	Brackish	3	3	60	nutrient limitation, growth rate
Boersma [4]	Chlorophyta	Tro	%, ug mg C^{-1}	Fresh	1	1	3	nutrient limitation
Broglio *et al.* [5]	Cryptophyta, Diatoms, Dinophyta	Tro	%	Marine	3	3	3	NA
Brown *et al.* [6]	Diatoms	Phy	%, ug mg C^{-1}	Marine	1	1	12	growth phase, light intensity
Chen *et al.* [7]	Cryptophyta, Diatoms, Haptophyta	Tro	%, ug mg C^{-1}	Marine	3	3	6	nutrient limitation
Chen [8]	Diatoms	Phy	%, %DW	Marine	1	12	12	NA (see supp. methods)
Chia *et al.* [9]	Chlorophyta	Phy	%	Fresh	1	1	9	nutrient limitation
Dunstan *et al.* [10]	Chlorophyta	Phy	%, ug mg DW^{-1}	Marine	1	7	9	NA
Dunstan *et al.* [11]	Eustigmatophyceae, Haptophyta	Phy	%, pg FA $cell^{-1}$	Marine	2	3	14	growth phase
Dunstan *et al.* [12]	Diatoms	Phy	%, pg FA $cell^{-1}$	Marine	1	14	14	NA
Dunstan *et al.* [13]	Cryptophyta, Rhodophyta	Phy	%, pg FA $cell^{-1}$	Marine	2	7	11	NA
Dutz *et al.* [14]	Cryptophyta, Diatoms, Dinophyta, Haptophyta	Tro	%^	Marine	4	10	11	NA
Fabregas *et al.* [15]	Eustigmatophyceae	Phy	%	Marine	1	1	5	light intensity
George *et al.* [16]	Chlorophyta, Haptophyta	Tro	%	Marine	2	2	2*	NA
Graeve *et al.* [17]	Diatoms, Dinophyta	Tro	%	Marine	2	2	2*	NA

Guedes *et al.* [18]	Chlorophyta, Cyanobacteria, Diatoms, Eustigmatophyceae, Haptophyta, Rhodophyta	Phy	%	Fresh and Marine	6	23	28	growth phase (only stationary)
Gugger *et al.* [19]	Cyanobacteria	Phy	%	Fresh	1	10	22	NA
Guihéneuf *et al.* [20]	Diatoms, Haptophyta	Phy	%	Marine	2	2	7	NA
Jónasdóttir [21]	Cryptophyta, Diatoms, Dinophyta	Tro	%	Marine	3	3	13*	growth phase
Jónasdóttir & Kiorboe [22]	Cryptophyta, Diatoms, Dinophyta	Tro	%^	Marine	3	7	14*	growth phase
Jónasdóttir *et al.* [23]	Chlorophyta, Diatoms, Dinophyta	Tro	%, pg FA cell^{-1}	Marine	3	5	5	NA
Kawachi *et al.* [24]	Pinguiophyceae	Phy	%	Marine	1	5	5*	NA
Liang *et al.* [25]	Diatoms	Phy	%	Marine	1	2	27	growth phase
Mansour *et al.* [26]	Dinophyta	Phy	%, %DW	Marine	1	5	10	NA
Marshall *et al.* [27]	Raphidophyceae	Phy	%, pg FA cell^{-1}	Marine	1	7	12	NA
Mooney *et al.* [28]	Dinophyta	Phy	%	Marine	1	8	11	NA
Mourente *et al.* [29]	Chlorophyta, Eustigmatophyceae, Haptophyta	Phy	%, %DW	Marine	3	11	12	NA
Müller-Navarra [30]	Chlorophyta, Cryptophyta, Diatoms	Tro	%, ug mg C^{-1}	Fresh	3	3	3	NA
Nichols *et al.* [31]	Dinophyta	Phy	%, %DW	Marine	1	2	2	NA
Nichols *et al.* [32]	Raphidophyceae	Phy	%	Marine	1	2	3	NA
Nichols *et al.* [33]	Haptophyta	Phy	%, pg FA cell^{-1}	Marine	1	1	7	NA
Parrish *et al.* [34]	Chlorophyta, Cryptophyta, Diatoms, Haptophyta	Tro	%	Marine	4	6	6*	NA
Patil *et al.* [35]	Chlorophyta, Cryptophyta, Cyanobacteria, Diatoms, Eustigmatophyceae, Haptophyta, Xanthophyceae	Phy	%, ug mg DW^{-1}	Fresh and Marine	7	12	12	NA
Pernet *et al.* [36]	Diatoms, Haptophyta	Phy	%	Marine	2	2	2	NA
Piepho *et al.* [37]	Chlorophyta, Cryptophyta, Diatoms	Phy	%, ug mg C^{-1}^	Fresh	3	4	56	nutrient limitation, light intensity
Pugh [38]	Diatoms	Phy	%	Brackish and Marine	1	1	8	growth phase, salinity

[39]	Chlorophyta, Diatoms, Dinophyta, Haptophyta	Phy	%, ug mg DW^{-1}	Marine	4	7	14	nutrient limitation (degrees of limitation)
Renaud *et al.* [40]	Diatoms, Haptophyta	Phy	%	Brackish	2	2	8	light intensity
Renaud *et al.* [41]	Chlorophyta, Cryptophyta, Diatoms, Haptophyta, Rhodophyta	Phy	%, %DW	Brackish	5	15	18	NA
Renaud *et al.* [42]	Cryptophyta, Diatoms, Haptophyta	Phy	%, %DW	Brackish	3	5	20	temperature
Rezanka *et al.* [43]	Cyanobacteria	Phy	%	Fresh	1	6	6	nutrient limitation (only limited)
Schwenk *et al.* [44]	Chlorophyta, Cyanobacteria, Diatoms, Dinophyta, Haptophyta	Phy	%, %DW	Brackish and Marine	5	17	35	nutrient limitation, growth phase, temperature, salinity
Skerratt *et al.* [45]	Diatoms, Haptophyta	Phy	%, pg FA $cell^{-1}$	Marine	2	3	9	UV radiation
Taipale *et al.* [46]	Chlorophyta, Chrysophyceae, Cryptophyta, Diatoms, Euglenozoa, Raphidophyceae	Phy	%	Fresh	6	35	37	NA
Tang *et al.* [47]	Cryptophyta, Haptophyta	Tro	%	Marine	2	2	2	NA
Thompson *et al.* [48]	Chlorophyta, Diatoms, Haptophyta	Phy	%	Marine	3	8	38	temperature
Thompson *et al.* [49]	Diatoms, Haptophyta	Tro	%	Marine	2	2	4	light intensity
Thor *et al.* [50]	Chlorophyta, Dinophyta, Haptophyta	Tro	%^	Marine	3	3	3	NA
Tremblay *et al.* [51]	Cryptophyta, Diatoms	Tro	%, pg FA $cell^{-1}$	Marine	2	3	3	NA
Vargas *et al.* [52]	Cyanobacteria	Phy	%, %DW	Fresh	1	7	12	NA
Veloza *et al.* [53]	Chlorophyta, Cryptophyta	Tro	%, ug mg C^{-1}	Marine	2	2	2*	NA
Viso & Marty [54]	Chlorophyta, Cryptophyta, Cyanobacteria, Diatoms, Dinophyta, Haptophyta, Raphidophyceae, Rhodophyta, Xanthophyceae	Phy	%, ug mg C^{-1}	Marine	9	28	28	NA

Volkman *et al.* [55]	Chlorophyta, Cryptophyta, Diatoms, Haptophyta	Phy	%, pg FA cell^{-1}	Marine	4	10	10	NA
Wacker *et al.* [56]	Chlorophyta, Cyanobacteria, Eustigmatophyceae, Haptophyta	Tro	%, ug mg POC^{-1}	Fresh and Marine	4	5	5	NA
Wenzel *et al.* [57]	Cryptophyta	Tro	%, ug mg C^{-1}	Fresh	1	1	1	NA
Zhukova & Aizdaicher [58]	Chlorophyta, Cryptophyta, Diatoms, Dinophyta, Haptophyta, Rhodophyta	Phy	%	Marine	6	15	15	NA

[1] Study type abbreviations are ‘Phy’ for phycological studies, and ‘Tro’ for trophic studies
[2] Data types: ‘%’ is percent of total FA (from papers that extracted FA from total lipids); ‘%DW’ is FA percent of algal dry weight.
[3] If a different number of profiles were present in each data set the group number, n Taxa, and n FA profiles is based on % total FA data
[4] The treatments and manipulated variables column summarizes additional experimental manipulations in some studies
~this is the number of unique species, with different names, in the study that made it through the screening protocol and into the master file (see S1 File Methods)

^authors provided additional raw data beyond what is shown in the original manuscript

*Studies with one environmental variable missing; these profiles could not be used in DISTLM analysis, but were included in calculating food quality index

References

1. Ackman RG, Tocher CS. Marine phytoplankter fatty acids. J Fish Res Board Can. 1968;25: 1603–1620.
2. Ahlgren G, Gustafsson IB, Boberg M. Fatty acid content and chemical composition of fresh-water microalgae. J Phycol. 1992;28: 37–50. doi:10.1111/j.0022-3646.1992.00037.x
3. Bi R, Arndt C, Sommer U. Linking elements to biochemicals: effects of nutrient supply ratios and growth rates on fatty acid composition of phytoplankton species. J Phycol. 2014;50: 117–130.
4. Boersma M. The nutritional quality of P-limited algae for *Daphnia*. Limnol Oceanogr. 2000;45: 1157–1161.
5. Broglio E, Jónasdóttir SH, Calbet A, Jakobsen HH, Saiz E. Effect of heterotrophic versus autotrophic food on feeding and reproduction of the calanoid copepod *Acartia tonsa*: relationship with prey fatty acid composition. Aquat Microb Ecol. 2003;31: 267–278.
6. Brown MR, Dunstan GA, Norwood SJ, Miller KA. Effects of harvest stage and light on the biochemical composition of the diatom *Thalassiosira pseudonana*. J Phycol. 1996;32: 64–73. doi:10.1111/j.0022-3646.1996.00064.x
7. Chen X, Wakeham SG, Fisher NS. Influence of iron on fatty acid and sterol composition of marine phytoplankton and copepod consumers. Limnol Oceanogr. 2011;56: 716–724. doi:10.4319/lo.2011.56.2.0716
8. Chen Y-C. The biomass and total lipid content and composition of twelve species of marine diatoms cultured under various environments. Food Chem. 2012;131: 211–219. doi:10.1016/j.foodchem.2011.08.062

9. Chia MA, Lombardi AT, Gama Melao MDG, Parrish CC. Effects of cadmium and nitrogen on lipid composition of *Chlorella vulgaris* (Trebouxiophyceae, Chlorophyta). Eur J Phycol. 2013;48: 1–11. doi:10.1080/09670262.2012.750687
10. Dunstan GA, Volkman JK, Jeffrey SW, Barrett SM. Biochemical composition of microalgae from the green algal classes Chlorophyceae and Prasinophyceae. 2. Lipid classes and fatty acids. J Exp Mar Biol Ecol. 1992;161: 115–134. doi:10.1016/0022-0981(92)90193-E
11. Dunstan GA, Volkman JK, Barret SM, Garland CD. Changes in the lipid composition and maximization of the polyunsaturated fatty acid content of three microalgae grown in mass culture. J Appl Phycol. 1993;5: 71–83.
12. Dunstan GA, Volkman JK, Barrett SM, Leroi JM, Jeffrey SW. Essential polyunsaturated fatty-acids from 14 species of diatom (Bacillariophyceae). Phytochemistry. 1994;35: 155–161.
13. Dunstan GA, Brown MR, Volkman JK. Cryptophyceae and Rhodophyceae; chemotaxonomy, phylogeny, and application. Phytochemistry. 2005;66: 2557–2570.
14. Dutz J, Koski M, Jónasdóttir SH. Copepod reproduction is unaffected by diatom aldehydes or lipid composition. Limnol Oceanogr. 2008;53: 225–235.
15. Fabregas J, Maseda A, Dominguez A, Otero A. The cell composition of *Nannochloropsis* sp changes under different irradiances in semicontinuous culture. World J Microbiol Biotechnol. 2004;20: 31–35. doi:10.1023/B:WIBI.0000013288.67536.ed
16. George SB, Fox C, Wakeham S. Fatty acid composition of larvae of the sand dollar *Dendraster excentricus* (Echinodermata) might reflect FA composition of the diets. Aquaculture. 2008;285: 167–173.
17. Graeve M, Kattner G, Hagen W. Diet induced changes in the fatty acid composition of arctic herbivorous copepods - experimental evidence of trophic markers. J Exp Mar Biol Ecol. 1994;182: 97–110. doi:10.1016/0022-0981(94)90213-5
18. Guedes AC, Amaro HM, Barbosa CR, Pereira RD, Malcata FX. Fatty acid composition of several wild microalgae and cyanobacteria, with a focus on eicosapentaenoic, docosahexaenoic and alpha-linolenic acids for eventual dietary uses. Food Res Int. 2011;44: 2721–2729. doi:10.1016/j.foodres.2011.05.020
19. Gugger M, Lyra C, Suominen I, Tsitko I, Humbert J-F, Salkinoja-Salonen MS, et al. Cellular fatty acids as chemotaxonomic markers of the genera *Anabaena*, *Aphanizomenon*, *Microcystis*, *Nostoc* and *Planktothrix* (cyanobacteria). Int J Syst Evol Microbiol. 2002;52: 1007–1015.
20. Guihéneuf F, Fouqueray M, Mimouni V, Ulmann L, Jacquette B, Tremblin G. Effect of UV stress on the fatty acid and lipid class composition in two marine microalgae *Pavlova lutheri* (Pavlovophyceae) and *Odontella aurita* (Bacillariophyceae). J Appl Phycol. 2010; doi: 10.1007/s10811–010–9503–0.
21. Jónasdóttir SH. Effects of food quality on the reproductive success of *Acartia tonsa* and *Acartia hudsonica*: laboratory observations. Mar Biol. 1994;121: 67–81.
22. Jónasdóttir SH, Kiorboe T. Copepod recruitment and food composition: do diatoms affect hatching success? Mar Biol. 1996;125: 743–750.
23. Jónasdóttir SH, Visser AW, Jesperson C. Assessing the role of food quality in the production and hatching of *Temora longicornis* eggs. Mar Ecol Prog Ser. 2009;382: 139–150.
24. Kawachi M, Inouye I, Honda D, O'Kelly CJ, Bailey JC, Bidigare RR, et al. The Pinguiophyceae classis nova, a new class of photosynthetic stramenopiles whose members produce large amounts of omega-3 fatty acids. Phycol Res. 2002;50: 31–47. doi:10.1046/j.1440-1835.2002.00260.x
25. Liang Y, Beardall J, Heraud P. Changes in growth, chlorophyll fluorescence and fatty acid composition with culture age in batch cultures of *Phaeodactylum tricornutum* and *Chaetoceros muelleri* (Bacillariophyceae). Bot Mar. 2006;49: 165–173. doi:10.1515/bot.2006.021

26. Mansour MP, Volkman JK, Jackson AE, Blackburn SI. The fatty acid and sterol composition of five marine dinoflagellates. J Phycol. 1999;35: 710–720. doi:10.1046/j.1529-8817.1999.3540710.x
27. Marshall J-A, Nichols PD, Hallegraeff GM. Chemotaxonomic survey of sterols and fatty acids in six marine raphidophyte algae. J Appl Phycol. 2002;14: 255–265.
28. Mooney BD, Nichols PD, de Salas MF, Hallegraeff GM. Lipid, fatty acid, and sterol composition of eight species of Kareniaceae (Dinophyta): chemotaxonomy and putative lipid phycotoxins. J Phycol. 2007;43: 101–111. doi:10.1111/j.1529-8817.2006.00312.x
29. Mourente G, Lubian LM, Odriozola JM. Total fatty acid composition as a taxonomic index of some marine microalgae used as food in marine aquaculture. Hydrobiologia. 1990;203: 147–154. doi:10.1007/bf00005683
30. Müller-Navarra DC. The nutritional importance of polyunsaturated fatty acids and their use as trophic markers for herbivorous zooplankton: does it contradict? Arch Hydrobiol. 2006;167: 501–513. doi:10.1127/0003-9136/2006/0167-0501
31. Nichols PD, Jones GJ, Deleeuw JW, Johns RB. The fatty-acid and sterol composition of 2 marine dinoflagellates. Phytochemistry. 1984;23: 1043–1047. doi:10.1016/s0031-9422(00)82605-9
32. Nichols PD, Volkman JK, Hallegraeff GM, Blackburn SI. Sterols and fatty acids of the red tide flagellates *Heteroslgma akashiwo* and *Chaltonella antiqua* (Raphidophyceae). Phytochemistry. 1987;26: 2537–2541.
33. Nichols PD, Skerratt JH, Davidson A, Burton H, Mcmeekin TA. Lipids of cultured *Phaeocystis pouchetii*: Signatures for food-web, biogeochemical and environmental studies in Antarctica and the Southern ocean. Phytochemistry. 1991;30: 3209–3214. doi:10.1016/0031-9422(91)83177-M
34. Parrish CC, French VM, Whiticar MJ. Lipid class and fatty acid composition of copepods (*Calanus finmarchicus*, *C. glacialis*, *Pseudocalanus* sp., *Tisbe furcata* and *Nitokra lacustris*) fed various combinations of autotrophic and heterotrophic protists. J Plankton Res. 2012;34: 356–375. doi:10.1093/plankt/fbs003
35. Patil V, Kallqvist T, Olsen E, Vogt G, Gislerod HR. Fatty acid composition of 12 microalgae for possible use in aquaculture feed. Aquac Int. 2007;15: 1–9. doi:10.1007/s10499-006-9060-3
36. Pernet F, Tremblay R, Demers E, Roussy M. Variation of lipid class and fatty acid composition of *Chaetoceros muelleri* and *Isochrysis* sp grown in a semicontinuous system. Aquaculture. 2003;221: 393–406. doi:10.1016/s0044-8486(03)00030-9
37. Piepho M, Arts MT, Wacker A. Species-specific variation in fatty acid concentrations of four phytoplankton species: does phosphorus supply influence the effect of light intensity or temperature? J Phycol. 2012;48: 64–73.
38. Pugh PR. Changes in the fatty acid composition of *Coscinodiscus eccentricus* with culture-age and salinity. Mar Biol. 1971;11: 118–124.
39. Reitan KI, Rainuzzo JR, Olsen Y. Effect of nutrient limitation on fatty acid and lipid content of marine microalgae. J Phycol. 1994;30: 972–979. doi:10.1111/j.0022-3646.1994.00972.x
40. Renaud SM, Parry DL, Thinh LV, Kuo C, Padovan A, Sammy N. Effect of light-intensity on the proximate biochemical and fatty acid composition of *Isochrysis* sp and *Nannochloropsis oculata* for use in tropical aquaculture. J Appl Phycol. 1991;3: 43–53. doi:10.1007/bf00003918
41. Renaud SM, Thinh LV, Parry DL. The gross chemical composition and fatty acid composition of 18 species of tropical Australian microalgae for possible use in mariculture. Aquaculture. 1999;170: 147–159.
42. Renaud SM, Thinh LV, Lambrinidis G, Parry DL. Effect of temperature on growth, chemical composition and fatty acid composition of tropical Australian microalgae grown in batch cultures. Aquaculture. 2002;211: 195–214.

43. Rezanka T, Dor I, Prell A, Dembitsky VM. Fatty acid composition of six freshwater wild cyanobacterial species. Folia Microbiol (Praha). 2003;48: 71–75. doi:10.1007/bf02931279
44. Schwenk D, Seppala J, Spilling K, Virkki A, Tamminen T, Oksman-Caldentey KM, et al. Lipid content in 19 brackish and marine microalgae: influence of growth phase, salinity and temperature. Aquat Ecol. 2013;47: 415–424. doi:10.1007/s10452-013-9454-z
45. Skerratt JH, Davidson AD, Nichols PD, McMeekin TA. Effect of EV-B on lipid content of three antarctic marine phytoplankton. Phytochemistry. 1998;4: 999–1007.
46. Taipale S, Strandberg U, Peltomaa E, Galloway AWE, Ojala A, Brett MT. Fatty acid composition as biomarkers of freshwater microalgae: analysis of 37 strains of microalgae in 22 genera and in seven classes. Aquat Microb Ecol. 2013;71: 165–178. doi:10.3354/ame01671
47. Tang KW, Jakobsen HH, Visser AW. *Phaeocystis globosa* (Prymnesiophyceae) and the planktonic food web: feeding, growth, and trophic interactions among grazers. Limnol Oceanogr. 2001;46: 1860–1870.
48. Thompson PA, Guo MX, Harrison PJ, Whyte JNC. Effects of variation in temperature on the fatty-acid composition of 8 species of marine-phytoplankton. J Phycol. 1992;28: 488–497.
49. Thompson PA, Guo M -x., Harrison PJ. Nutritional value of diets that vary in fatty acid composition for larval Pacific oysters (*Crassostrea gigas*). Aquaculture. 1996;143: 379–391.
50. Thor P, Koski M, Tang KW, Jónasdóttir SH. Supplemental effects of diet mixing on absorption of ingested organic carbon in the marine copepod *Acartia tonsa*. Mar Ecol Prog Ser. 2007;331: 131–138. doi:10.3354/meps331131
51. Tremblay R, Cartier S, Miner P, Pernet F, Quere C, Moal J, et al. Effect of *Rhodomonas salina* addition to a standard hatchery diet during the early ontogeny of the scallop *Pecten maximus*. Aquaculture. 2007;262: 410–418.
52. Vargas MA, Rodriguez H, Moreno J, Olivares H, Del Campo JA, Rivas J, et al. Biochemical composition and fatty acid content of filamentous nitrogen-fixing cyanobacteria. J Phycol. 1998;34: 812–817. doi:10.1046/j.1529-8817.1998.340812.x
53. Veloza AJ, Chu FL, Tang KW. Trophic modification of essential fatty acids by heterotrophic protists and its effects on the fatty acid composition of the copepod *Acartia tonsa*. Mar Biol. 2006;148: 779–788.
54. Viso AC, Marty JC. Fatty acids from 28 marine microalgae. Phytochemistry. 1993;34: 1521–1533. doi:10.1016/s0031-9422(00)90839-2
55. Volkman JK, Jeffrey SW, Nichols PD, Rogers GI, Garland CD. Fatty-acid and lipid-composition of 10 species of microalgae used in mariculture. J Exp Mar Biol Ecol. 1989;128: 219–240.
56. Wacker A, Becher P, von Elert E. Food quality effects of unsaturated fatty acids on larvae of the zebra mussel *Dreissena polymorpha*. Limnol Oceanogr. 2002;47: 1242–1248.
57. Wenzel A, Bergstrom AK, Jansson M, Vrede T. Survival, growth and reproduction of *Daphnia galeata* feeding on single and mixed *Pseudomonas* and *Rhodomonas* diets. Freshw Biol. 2012;57: 835–846. doi:10.1111/j.1365-2427.2012.02751.x
58. Zhukova NV, Aizdaicher NA. Fatty acid composition of 15 species of marine microalgae. Phytochemistry. 1995;39: 351–356.
