# Supplementary material for: Partitioning the Relative Importance of Phylogeny and Environmental Conditions on Phytoplankton Fatty Acids
Source: PLoS One. 2015 Jun 15;10(6):e0130053. doi: 10.1371/journal.pone.0130053 (PMC4468072; doi:10.1371/journal.pone.0130053)
Supplement: S2 Table — Table is organized by FA data type and indicating the six major algal groups that are the focus of analyses. The FA observation (n profiles) in the ‘included’ category is the number of raw profiles that were used in the PCA (S1 Fig) or eligible for inclusion in the DISTLM analysis. (PDF) [file pone.0130053.s006.pdf]

S2 Table. Number of FA profiles in each of the algal groups in the full meta-analysis data set. Table is organized by FA data type and indicating the six major algal groups that are the focus of analyses. The FA observation (n profiles) in the 'included' category is the number of raw profiles that were used in the PCA (S1 Fig.) or eligible for inclusion in the DISTLM analysis.

	% FA	FA % DW
Algal groups	n profiles	n profiles
Included		
Chlorophyta	129	16
Cryptophyta	87	9
Cyanobacteria	76	14
Diatoms	232	43
Dinophyta	41	10
Haptophyta	101	17
Excluded		
Chrysophyceae	6	0
Euglenozoa	3	0
Eustigmatophyceae	25	6
Pinguiophyceae	5	0
Raphidophyceae	28	0
Rhodophyta	7	1
Xanthophyceae	2	0
