# Supplementary material for: Partitioning the Relative Importance of Phylogeny and Environmental Conditions on Phytoplankton Fatty Acids
Source: PLoS One. 2015 Jun 15;10(6):e0130053. doi: 10.1371/journal.pone.0130053 (PMC4468072; doi:10.1371/journal.pone.0130053)
Supplement: S3 Table — Results of DISTLM marginal tests, quantifying the relative contribution of algal group affiliation and culture condition variables (following abbreviations of Table 1) for both fatty acid datasets (% FA and FA % DW). The marginal tests result reports the proportion of the variation (prop. var.) explained by each variable, independent of any others (see Methods). (PDF) [file pone.0130053.s007.pdf]

S3 Table. Results of DISTLM marginal tests. Results of marginal tests using a distance-based linear model (DISTLM) quantifying the relative contribution of algal group affiliation and culture condition variables [nutrient status (e.g., replete and limited; Nutrient), light intensity (Light), hours of light (Hrs. Light), temperature (Temp), and salinity] for both fatty acid datasets (% FA and FA % DW). The marginal tests result reports the proportion of the variation (prop. var.) explained by each variable, independent of any others (see Methods).

		% FA				FA % DW			
Variable	reg. df	Pseudo -*F*	*P*	prop. var.	res. df	Pseudo -*F*	*P*	prop. var.	res. df
Group	6	95.16	0.0001	0.436	615	11.67	0.0001	0.362	103
Nutrient	2	4.56	0.0006	0.007	619	6.40	0.0008	0.056	107
Light	2	9.03	0.0001	0.014	619	8.23	0.0003	0.071	107
Hrs. Light	2	21.39	0.0001	0.033	619	13.52	0.0001	0.112	107
Temp	2	8.37	0.0001	0.013	619	3.41	0.0097	0.031	107
Salinity	2	76.53	0.0001	0.110	619	6.02	0.0003	0.053	107
